# Language distance in orthographic transparency affects cross‐language pattern similarity between native and non‐native languages

**DOI:** 10.1002/hbm.25266

**Published:** 2020-10-28

**Authors:** Jie Dong, Aqian Li, Chuansheng Chen, Jing Qu, Nan Jiang, Yue Sun, Liyuan Hu, Leilei Mei

**Affiliations:** ^1^ Key Laboratory of Brain Cognition and Education Sciences (South China Normal University), Ministry of Education Guangzhou China; ^2^ School of Psychology South China Normal University Guangzhou China; ^3^ Center for Studies of Psychological Application South China Normal University Guangzhou China; ^4^ Guangdong Key Laboratory of Mental Health and Cognitive Science South China Normal University Guangzhou China; ^5^ Department of Psychological Science University of California Irvine California USA

**Keywords:** bilingual, fMRI, orthographic transparency, pattern similarity, word reading

## Abstract

How native and non‐native languages are represented in the brain is one of the most important questions in neurolinguistics. Much research has found that the similarity in neural activity of native and non‐native languages are influenced by factors such as age of acquisition, language proficiency, and language exposure in the non‐native language. Nevertheless, it is still unclear how the similarity between native and non‐native languages in orthographic transparency, a key factor that affects the cognitive and neural mechanisms of phonological access, modulates the cross‐language similarity in neural activation and which brain regions show the modulatory effects of language distance in orthographic transparency. To address these questions, the present study used representational similarity analysis (RSA) to precisely estimate the neural pattern similarity between native language and two non‐native languages in Uyghur‐Chinese‐English trilinguals, whose third language (i.e., English) was more similar to the native language (i.e., Uyghur) in orthography than to their second language (i.e., Chinese). Behavioral results revealed that subjects responded faster to words in the non‐native language with more similar orthography to their native language in the word naming task. More importantly, RSA revealed greater neural pattern similarity between Uyghur and English than between Uyghur and Chinese in select brain areas for phonological processing, especially in the left hemisphere. Further analysis confirmed that those brain regions represented phonological information. These results provide direct neuroimaging evidence for the modulatory effect of language distance in orthographic transparency on cross‐language pattern similarity between native and non‐native languages during word reading.

## Graphical abstract

Greater cross‐language pattern similarity was associated with a smaller language distance in orthographic transparency in brain areas for phonological processing, especially in the left hemisphere. Further analysis confirmed that those brain regions represented phonological information. This study provides direct neuroimaging evidence for the modulatory effect of language distance in orthographic transparency on cross‐language pattern similarity.

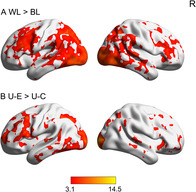

## 1 INTRODUCTION

Bilingual research has become a hot topic since a large proportion of the population has acquired two or more languages in this globalization era (Blanco‐Elorrieta & Pylkkanen, 2018). One of the most important questions in this area is how one brain processes multiple languages. Numerous neuroimaging studies have found that bilinguals recruit similar brain regions including the prefrontal cortex, temporoparietal cortex, and occipitotemporal regions, to read in native and non‐native languages (Cao, Tao, Liu, Perfetti, & Booth, 2013; Kim et al., 2016; Kim, Liu, & Cao, 2017; Nakada, Fujii, & Kwee, 2001; Tan et al., 2003; van de Putte, de Baene, Brass, & Duyck, 2017). This phenomenon is more pronounced at the single‐word level than at the sentence level (Briellmann et al., 2004; Li et al., 2019; Mei, Xue, Lu, Chen, et al., 2015; Nelson, Liu, Fiez, & Perfetti, 2009; Xue, Dong, Jin, Zhang, & Wang, 2004). Furthermore, the degree of similarity in activation patterns between native and non‐native languages has been found to be affected by language proficiency (Bowden, Steinhauer, Sanz, & Ullman, 2013; Cao et al., 2013; Gao et al., 2017; Kim et al., 2017; Li et al., 2019; Rossi, Gugler, Friederici, & Hahne, 2006; Sun et al., 2015), age of acquisition (Berken et al., 2015; Chee, Tan, & Thiel, 1999; Das, Padakannaya, Pugh, & Singh, 2011; Gathercole & Moawad, 2010; Jasinska & Petitto, 2013), and language exposure in non‐native languages (Perani et al., 2003; Tu et al., 2015).

In addition to the abovementioned factors, language distance in orthographic transparency (i.e., the regularity of mapping from graphemes to phonemes) (Liu & Cao, 2016) may also affect the similarity in activation patterns between native and non‐native languages. Cognitive models of reading have proposed that the phonological form of a word can be accessed either using information about how letters correspond to sounds, or via the orthographic and phonological lexicons (dual route cascaded model) or the semantic system (triangle model) (Coltheart, Rastle, Perry, Langdon, & Ziegler, 2001; Harm & Seidenberg, 2004; Plaut, Mcclelland, Seidenberg, & Patterson, 1996). Researchers have further revealed that orthographic transparency modulates the extent to which these two pathways are used to derive the pronunciation of a word from its written form (Bigozzi, Tarchi, & Pinto, 2016; Coltheart et al., 2001; Dehaene, Cohen, Sigman, & Vinckier, 2005; Meschyan & Hernandez, 2006; Simon, Bernard, Lalonde, & Rebai, 2006), and consequently influences the recruitment of brain regions in word reading (Hartwigsen et al., 2016; Jobard, Crivello, & Tzourio‐Mazoyer, 2003; Mei, Xue, Lu, Chen, et al., 2015; Miozzo, Williams, McKhann, & Hamberger, 2017; Pillay, Stengel, Colin, Book, & Binder, 2015; Price, 2012; Taylor, Rastle, & Davis, 2013). Specifically, reading words in transparent orthography with a regular grapheme‐to‐phoneme correspondence (GPC) rule (e.g., Italian) relies more on the orthography‐to‐phonology mapping pathway (Nosarti, Mechelli, Green, & Price, 2010; Paulesu et al., 2000) and hence shows activations in brain regions for phonological processing such as the left precentral gyrus (PCG), dorsal inferior frontal gyrus and temporoparietal cortex (Chen, Xue, Mei, Chen, & Dong, 2009; Cummine et al., 2013; Jobard et al., 2003; Mei, Xue, Lu, He, et al., 2015; Price, 2012). The left PCG is thought to be related to overt articulation in word reading (Niu, Nie, Zhou, Zhu, & Wei, 2016; Price, 2012). The left dorsal inferior frontal gyrus is thought to be responsible for syllabification in speech production and the left temporoparietal cortex (e.g., the supramarginal gyrus [SMG] and posterior superior temporal gyrus [pSTG]) plays an important role in GPC during word reading (Booth et al., 2006; Fiez, Raichle, Balota, Paula, & Petersen, 1996; Howard et al., 1992; Price, 2012; Tan, Laird, Li, & Fox, 2005; Warburton et al., 1996). In contrast, reading words in nontransparent orthography whose visual forms map onto the whole syllable (e.g., Chinese) depends more on the semantics‐mediated pathway (Chen et al., 2009; Mei, Xue, Lu, He, et al., 2015; Tan et al., 2005) and hence elicits activation in brain regions for semantic processing, such as the left ventral inferior frontal gyrus, and lateral temporal cortex (Buetler et al., 2014; Cummine et al., 2013; Ischebeck et al., 2004; Taylor et al., 2013). The left ventral inferior frontal gyrus is associated with strategic semantic processing (Adams & Janata, 2002; Price, 2012). The middle and inferior temporal gyrus are thought to represent semantic information during word reading (Binder, Desai, Graves, & Conant, 2009; Miozzo et al., 2017; Price, 2000). Therefore, native and non‐native languages with small language distance in orthographic transparency are more likely to recruit the same phonological access pathway and consequently elicit similar neural activation in brain regions for word reading.

To our knowledge, only one study has explored the impact of language distance in orthographic transparency on the similarity of neural activation between native and non‐native languages (Kim et al., 2016). In that study, a visual rhyming judgment task was conducted to detect neural activity in Korean‐Chinese‐English trilinguals. By calculating the degree of similarity between native and non‐native languages (i.e., the proportion of overlapped activation volume out of the total activation volume in the two languages), they found that native and non‐native languages with a small distance in orthographic transparency (i.e., Korean and English) showed more similar activation than those with a large distance in orthographic transparency (i.e., Korean and Chinese). These findings suggest that the similarity of neural activation between native and non‐native languages is modulated by language distance in orthographic transparency.

Nevertheless, Kim et al.'s study has at least three limitations. First, the similarity index was calculated based on the results of a univariate activation analysis, which treated each voxel independently and consequently missed fine‐grained pattern information (Haxby, 2012; Haynes, 2015). In contrast, multivariate methods (e.g., representational similarity analysis [RSA]) compute patterns of neural activity across multiple voxels, which are able to detect fine‐grained pattern differences even if there are no regional‐average differences (Mur, Bandettini, & Kriegeskorte, 2009). There is evidence that words in native and non‐native languages are differentially represented even in the brain regions showing similar activations for the two languages (Xu, Baldauf, Chang, Desimone, & Tan, 2017). Second, they used the overall proportion of overlapped activation as the index of activation similarity for two languages. Therefore, it is not clear which brain areas show the modulatory effects of language distance in orthographic transparency on the activation similarity between native and non‐native languages. Finally, the information represented in brain regions showing cross‐language pattern similarity was not addressed in Kim et al.'s study. By correlating neural pattern similarity matrices with visual, phonological, and semantic prediction matrices, RSA is able to disentangle the contributions of different linguistic information (e.g., orthographic, phonological, and semantic information) to similar activation patterns between native and non‐native languages. Thus, RSA is needed for a quantitative estimation of the neural pattern similarity between native and non‐native languages and for further exploration of information representation in brain regions showing cross‐language pattern similarity (Li et al., 2019).

To explore the modulatory effects of language distance in orthographic transparency on cross‐language pattern similarity, we recruited a group of Uyghur‐Chinese‐English trilingual individuals whose two non‐native languages (i.e., Chinese and English) differed from the native language (i.e., Uyghur) in orthographic transparency. Uyghur belongs to the Turkic language family, which is spoken by Uyghurs in the Xinjiang Uygur Autonomous Region of China. Uyghur is a transparent orthography because its pronunciation strictly conforms to the GPC rules (e.g., **ش** in شام /ʃam/ maps to /ʃ/; **ت** in تام /tam/maps to /t/) (Jiang et al., 2015; Zhao, Zhang, Chen, Zhou, & Zuo, 2016). Therefore, both Uyghur and English are alphabetic languages, which have a regular/semiregular alphabetic principle when converting graphemes to phonemes, whereas Chinese is a logographic language that has no letter‐phoneme mapping rules (Chen et al., 2009; Perfetti & Tan, 2013; Tan et al., 2005; Zhao et al., 2016; Ziegler & Goswami, 2005). As Chinese (logographic script) is more opaque than English and Uyghur (alphabetic script) in terms of orthographic transparency (Chen et al., 2009; Kim et al., 2016; Liu & Cao, 2016), the language distance between Uyghur and English is smaller than that between Uyghur and Chinese.

To ensure that subjects were highly engaged in phonological access, a word naming task was performed during the fMRI scan. Whole‐brain RSA was first used to explore all potential brain areas showing the modulatory effects of language distance in orthographic transparency on the neural pattern similarity between native and non‐native languages. Because the two languages with smaller language distances (i.e., Uyghur and English) are thought to heavily rely on the orthography‐to‐phonology mapping pathway (Chen et al., 2009; Cummine et al., 2013; Jobard et al., 2003; Mei, Xue, Lu, He, et al., 2015; Nosarti et al., 2010; Paulesu et al., 2000), ROI‐based RSA was then conducted to examine whether the effect of language distance occurred in the brain areas for phonological processing, including the bilateral (PCG, pars opercularis (PO), angular gyrus (AG), pSTG, and SMG (Chen et al., 2009; Cummine et al., 2013; Jobard et al., 2003; Mei, Xue, Lu, He, et al., 2015; Price, 2012). Finally, neural dissimilarity matrices were separately correlated with visual, phonological, and semantic prediction matrices to further examine information representation in brain regions showing the effect of language distance. We expected that greater cross‐language neural pattern similarity between Uyghur and English relative to those between Uyghur and Chinese would be found in the brain areas for phonological processing.

## 2 MATERIALS AND METHODS

### 2.1 Subjects

Twenty‐three Uyghur‐Chinese‐English trilinguals (17 females, aged 20–23 years) participated in the present study. The number of subjects was sufficient for investigating the effects of language distance according to the following two analyses. First, following the methods in Ueno, Meteyard, Hoffman, and Murayama (2018), we used Google Scholar to retrieve 25 neuroimaging studies on bilingualism published from January 2015 to March 2020. Among those 25 articles, 6 that investigated language processing in bilinguals by using a within‐subject design were directly related to our research. A random‐effects meta‐analysis of the six articles resulted in an integrated effect size of Cohen's *d* = 1.651 (confidence interval: 1.270–2.032). A power analysis on this integrated effect size revealed that a sample size of 23 would provide a power value of more than 0.999. Second, we used G*Power to estimate the appropriate sample size needed for our design. To detect a medium effect size (i.e., 0.25) with 0.80 power in repeated‐measures analysis of variance (ANOVA; Cohen, 1988, 1992), the expected sample size was 17 subjects.

Participants' native language was Uyghur, they started to learn written Uyghur at the mean age of 5 years (*SD* = 0.80) and continued until the end of junior high school, for an average of 7 years (*SD* = 2.22) of formal Uyghur language education. Subjects started to learn Chinese (Mandarin) at 9 years of age (*SD* = 2.71) and English at 15 years of age (*SD* = 1.80), and by the time of the experiment, they had received formal Chinese and English language education for 12 years (*SD* = 2.78) and 8 years (*SD* = 2.08), respectively. To determine their proficiency in the three languages, we asked subjects to self‐evaluate their proficiency on a 7‐point scale (1 = “quite poor,” 7 = “highly proficient”). The scores on Uyghur, Chinese, and English were 5.79 (*SD* = 0.89), 4.74 (*SD* = 0.52), and 3.55 (*SD* = 0.45), respectively. The proficiency level was higher for Uyghur than Chinese (*F*(1,22) = 56.28, *p* < .001), which was higher than English (*F*(1,22) = 99.31, *p* < .001). All subjects had normal or corrected‐to‐normal vision, and were right‐handed (Snyder & Harris, 1993). They did not suffer from any neurological or psychiatric disorders. Before the experiment, all subjects provided informed consent. This research was approved by the Institutional Review Board of the School of Psychology at South China Normal University.

### 2.2 Materials

**FIGURE 1:**
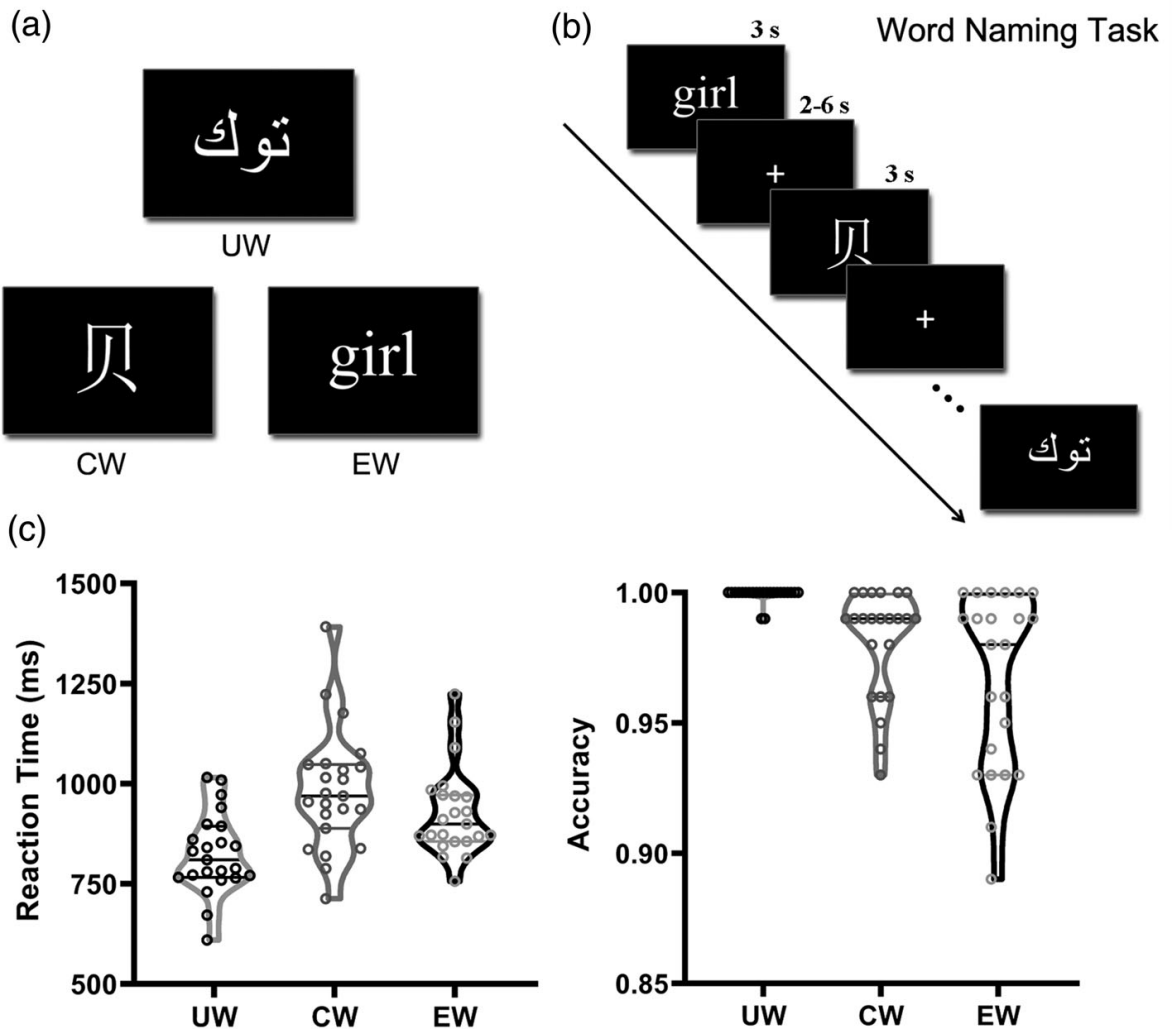
Experimental materials, the fMRI task, and behavioral performance. Uyghur words (UW), Chinese words (CW), and English words (EW) were used in this study (a). Subjects were scanned while performing a word naming task (b). For both reaction time and accuracy, subjects performed better for words in their native language than those in non‐native languages (c). Violin plots show the spread and differences in response times and accuracy for the three types of words

The materials of this study comprised 80 Uyghur words, 80 Chinese words, and 80 English words (see Table S6). All words were nouns (Figure 1a). They were presented in black‐scale with 226 × 151 pixels in size. Uyghur words were selected from Uyghur everyday vocabulary and were 3–6 letters in length (*M* = 4.41, *SD* = 1.11). The Chinese and English words were all medium‐ to high‐frequency words (Chinese words: *M* = 128.03 per million, *SD* = 83.00; English words: *M* = 98.85 per million, *SD* = 82.08) (Brysbaert & New, 2009; Cai & Brysbaert, 2010). The Chinese words were all single‐character words and consisted of four to eight strokes (*M* = 6.60, *SD* = 1.00). The English words consisted of three to six letters (*M* = 4.43, *SD* =1.10), which were matched with Uyghur words in length. All Uyghur and English words were regular words whose pronunciations complied with GPC rules. The pronunciation regularity of English words (a measure of spelling‐sound consistency) was further quantified by calculating the probabilities of graphemes being pronounced as their corresponding phonemes for the 80 English words used in this study (Berndt, Reggia, & Mitchum, 1987; Gontijo, Gontijo, & Shillcock, 2003). Specifically, we first calculated the pronunciation regularity of each grapheme of a word (e.g., “put”) by dividing the probability of the corresponding pronunciation of the grapheme (e.g., the grapheme “p” in “put” is pronounced as /p/, and its pronunciation probability is 1) by the probability of its most likely pronunciation (e.g., the most likely pronunciation of grapheme “p” is /p/, and its pronunciation probability is 1). Higher values of regularity (theoretical range = 0–1.0) indicate higher spelling‐sound consistency. The regularities for all the graphemes of a word were then averaged to yield the regularity for the word. The mean value of pronunciation regularity for all 80 English words was 0.88 (*SD* = 0.15), suggesting a high level of spelling‐sound consistency.

To avoid the potential cross‐language semantic priming effects, no translation equivalents across the three languages were used in this study. To ensure that subjects were familiar with the materials, we recruited another 14 Uyghur‐Chinese‐English trilinguals, who did not participate in the main experiment to assess the familiarity of all words on a 5‐point scale (1 = “very unfamiliar,” 5 = “very familiar”). The mean scores were 4.81 (*SD* = 0.15), 4.71 (*SD* = 0.20), and 4.62 (*SD* =0.28) for Uyghur, Chinese, and English words, respectively. These results indicate that the materials used in this study were familiar to the subjects.

### 2.3 fMRI task

A word naming task was performed during the fMRI scan. The experimenter, a female Chinese‐English bilingual, introduced the experiment to subjects in Chinese (Mandarin). To ensure that subjects understood the experimental instruction, they were asked to practice with a simplified version of the experiment task before the fMRI scan. Subjects were not allowed to participate in the formal experiment until their accuracy reached 100% in the practice session. In the naming task, subjects were instructed to read the words overtly (Figure 1b). During the task, the Uyghur, Chinese, and English words were pseudorandomly presented. To improve the efficiency of the design, we optimized trial sequences by using OPTSEQ2 (http://surfer.nmr.mgh.harvard.edu/optseq2/) (Dale, 2010).

Subjects were scanned in two functional runs. Each run included 120 trials with 40 trials for each language. Half of the materials in each language were presented in the first run, and the other half were presented in the second run. In each trial, a visual word was presented for 3 s, followed by a fixation that varied randomly from 2 to 6 s (*M* = 3 s). Subjects were asked to read the word as fast and accurately as possible. In total, each run lasted for 720 s. Before scanning, subjects practiced the simplified version of the fMRI task to familiarize themselves with the scanning procedure. Words used in the practice session were not presented during the main experiment. To further verify whether subjects actually read the words in the scanner, we monitored their vocal responses using an external audio device which was compatible with the MRI scanner. Due to the large amount of noise during scanning, the subjects' behavioral responses (i.e., accuracy and reaction time) of the naming task were recorded after scanning. The trial sequence and other experimental parameters were exactly the same as those of the fMRI task.

### 2.4 fMRI data acquisition

A 3.0 T Siemens MRI scanner was used for data collection in the MRI Center at South China Normal University. The functional imaging data were collected using a single‐shot T2*‐weighted gradient‐echo EPI sequence. The following scanning parameters were used: TR = 2,000 ms, TE = 30 ms, flip angle = 90°, FOV = 224 × 224 mm, matrix size = 64 × 64, slice thickness = 3.5 mm, and number of slices = 32. Anatomical data were collected with a T1‐weighted, gradient‐echo pulse sequence. The following parameters were used: TR = 1,900 ms, TE = 2.52 ms, flip angle = 9°, FOV = 256 × 256 mm, matrix size = 256 × 256, slice thickness = 1 mm, and number of slices = 176.

### 2.5 fMRI data preprocessing and analysis

FEAT (FMRI Expert Analysis Tool) Version 6.00 was used to process the imaging data. The first three images in each run were deleted to allow for T1 equilibrium effects. The rest of the functional images were then realigned and normalized to the Montreal Neurological Institute template (Jenkinson & Smith, 2001). The functional data were then spatially smoothed with a 5 mm full‐width at half‐maximum Gaussian kernel, and temporally filtered by using a nonlinear high‐pass filter with a 60 s cut‐off. The translational movement parameters were no more than 3 mm in any direction for any subject or run.

At the first level, general linear models were used to model the preprocessed data for each subject and for each run. A canonical hemodynamic response function (double‐gamma) was used to convolve with the onsets and durations of events. To improve statistical sensitivity, we also included six motion parameters as covariates in the analysis. Fixation was used as a baseline. The contrast images for the three types of words and their comparisons were calculated for each subject and each run.

For each subject, the data were concatenated across the two runs in the second‐level analysis by using a fixed‐effects model. For the group analysis (the third‐level models), a random‐effects model with FLAME Stage 1 only was used (Beckmann, Jenkinson, & Smith, 2003; Woolrich, 2008). All reported results were thresholded with a height threshold of *Z* > 3.1 and a cluster probability of *p* < .05. The Gaussian random field theory was used to correct the whole‐brain multiple comparisons (Worsley, 2010).

### 2.6 Representational similarity analysis

In this analysis, the unsmoothed data were used to construct the first‐level models mentioned above. Indeed, the only difference in preprocessing between the univariate analysis and the RSA was the unsmoothed data of the latter (Li et al., 2019; Taylor, Davis, & Rastle, 2019; Xu et al., 2017). Two types of RSA were then performed on the T‐statistics maps. One RSA was used to quantify the neural pattern similarity between native and non‐native languages, and the other one was used to associate neural dissimilarity matrices with visual, phonological, and semantic prediction matrices.

We calculated cross‐language pattern similarity by performing both whole‐brain RSA and ROI (region of interest)‐based RSA. In the whole‐brain RSA, we used a searchlight‐based method (Li et al., 2019; Xue et al., 2013). In this analysis, we extracted the activation patterns from a cubic region (125 voxels) centered on the target voxel for Uyghur, Chinese, and English words in each run (Kriegeskorte, Goebel, & Bandettini, 2006). Pearson correlation analysis was used to calculate pattern similarity between Uyghur and Chinese words and that between Uyghur and English words in each run. The correlation coefficients were transformed into Fisher's z scores, which were then averaged across the two runs. To verify the validity of the RSA in this study, we also compared within‐language with between‐language pattern similarity. Those two pattern similarities were computed by extracting the activation patterns for each condition and for each run and then correlating the activation patterns for within‐ or cross‐language pairs across the two runs (Li et al., 2019).

In the ROI‐based RSA, 10 brain areas responsible for phonological processing (Binder et al., 2009; Jobard et al., 2003; Price, 2012; Taylor et al., 2013) were anatomically defined as ROIs based on the Harvard‐Oxford atlas. As discussed in the Introduction, the ROIs consisted of the PCG, PO, AG, pSTG, and SMG in both the left and right hemispheres. As in the whole‐brain RSA, cross‐language pattern similarity between native and non‐native languages was calculated by using Pearson correlation analysis within each ROI and was then transformed into Fisher's z‐scores (Li et al., 2019; Xue et al., 2013). The effects of language distance were examined by comparing the two cross‐language pattern similarity scores.

To disentangle the contributions of different linguistic information (e.g., orthographic, phonological, and semantic information) to the effects of language distance found in the above analysis, we performed an additional RSA to associate neural dissimilarity matrices with visual, phonological, and semantic prediction matrices. In this analysis, T‐statistic maps were generated for the contrast of each item relative to baseline in each run, which created 80 statistical maps for each language (Taylor et al., 2019). Based on the T‐statistic maps, we then calculated the cross‐language neural dissimilarity matrix for each cross‐language pair (i.e., Uyghur‐English and Uyghur‐Chinese). Specifically, for each subject, within each ROI, we extracted the multivoxel response patterns from T‐maps for each of the 240 items (Kriegeskorte, Mur, & Bandettini, 2008). For each cross‐language pair, we then constructed an 80 × 80 neural dissimilarity matrix, in which each cell represented the Pearson correlation of the voxel‐wise T statistic for each pair of cross‐language items in a given ROI. The z‐scores were then computed by using Fisher's z transformation.

In addition to the neural dissimilarity matrix, we also calculated three prediction matrices, which respectively captured visual, phonological, and semantic dissimilarity for each cross‐language pair (see Figure S2). Specifically, for the visual prediction matrix, a binary silhouette of each word was used to compute the pixel‐wise nonoverlap regions of the two images for each cross‐language pair (Fischer‐Baum, Bruggemann, Gallego, Li, & Tamez, 2017; Kriegeskorte et al., 2008). For the phonological prediction matrix, we used the second coding scheme (i.e., vowel‐centric, L‐R) from the MatchCalculator tool, which developed by Colin Davis (www.pc.rhul.ac.uk/staff/c.davis/Utilities/MatchCalc/). It was calculated as 1 minus the proportion of same‐position phonemes shared in each cross‐language pair (Taylor et al., 2019). For example, the Uyghur‐English pair of تام /tɑm/ and team /ti:m/ have a one‐third phoneme dissimilarity. The Uyghur‐Chinese pair of تام /tɑm/ and 厅 /ting/ have dissimilarities of three‐fourth and two‐third phonemes when the tonal information was included and excluded, respectively (Fromkin, 1980; Moser, 1991). The semantic prediction matrix was estimated by dividing the words in the 3 languages into 12 categories according to their semantic similarity, including human, animal, plants, and so forth (Taylor et al., 2019). Item pairs from the same semantic category were denoted as 0, and pairs from different categories were denoted as 1. Finally, we calculated Spearman correlations between neural dissimilarity matrices and the three prediction matrices (i.e., visual, phonological, and semantic prediction matrices) in the ROIs showing a significant effect of language distance for each cross‐language pair. Permutation tests were conducted to examine the significance level of the Spearman correlations. Specifically, the neural dissimilarity matrix in each ROI was correlated with three predicted dissimilarity matrices of each cross‐language pair, which was permuted 5,000 times. These correlation coefficients were used to construct a distribution for each ROI and for each cross‐language pair. A nonparametric statistical test was obtained by calculating the proportion of randomized test statistics that exceeded the observed statistics (Zhao et al., 2017).

## 3 RESULTS

### 3.1 Behavioral results

We used one‐way repeated measures ANOVA to investigate the behavioral differences (i.e., reaction time and accuracy) across the three types of materials (Figure 1c). For both reaction time and accuracy, the main effects of language were significant (reaction time: *F*(2,44) = 29.27, *p* < .001; accuracy: *F*(2,44) = 13.11, *p* < .001). Post hoc comparisons revealed that for reaction time, Uyghur words (824.58 ms) were named faster than English words (926.87 ms) (*p* < .001), which were named faster than Chinese words (982.33 ms) (*p* < .01). Consistently, regarding accuracy, Uyghur words (99.89%) had higher accuracy levels than Chinese words (97.99%) (*p* < .001), which had slightly higher accuracy levels than English words (96.52%) (*p* = .055). These results suggest that subjects were more familiar with words in their native language (i.e., Uyghur words) than those in non‐native languages (i.e., Chinese and English words). In addition, subjects responded faster to the non‐native language with more similar orthography to their native language in the word naming task.

To rule out the possibility that the faster naming speed for English words relative to Chinese words was caused by the speed‐accuracy trade‐off, we performed two additional analyses. First, we combined reaction time and accuracy into a single measure (i.e., the inverse efficiency score) (Townsend & Ashby, 1983), which is defined as the mean reaction time divided by accuracy (Akhtar & Enns, 1989; Bruyer & Brysbaert, 2011). The results showed that the difference in the inverse efficiency score between English and Chinese words was marginally significant (*t*(22) = −1.94, *p* = .06). Second, we compared the reaction time of Chinese words with that of English words in 13 subjects with relatively high proficiency in English, whose accuracy did not differ between Chinese (98.17%) and English words (98.37%) (*t*(12) = −0.21, *n*.*s*.). We found that English words (909.40 ms) were still named significantly faster than Chinese words (977.95 ms) (*t*(12) = 2.44, *p* < .05). These two lines of evidence suggest that the faster naming speed for English words relative to Chinese words was not caused by the speed‐accuracy trade‐off but by their distance in orthographic transparency to the subject's native language (Hamada & Koda, 2008; Leikin, Share, & Schwartz, 2005; Mei, Xue, Lu, He, et al., 2015).

### 3.2 Neural activations for Uyghur, Chinese, and English words during the word naming task

**FIGURE 2:**
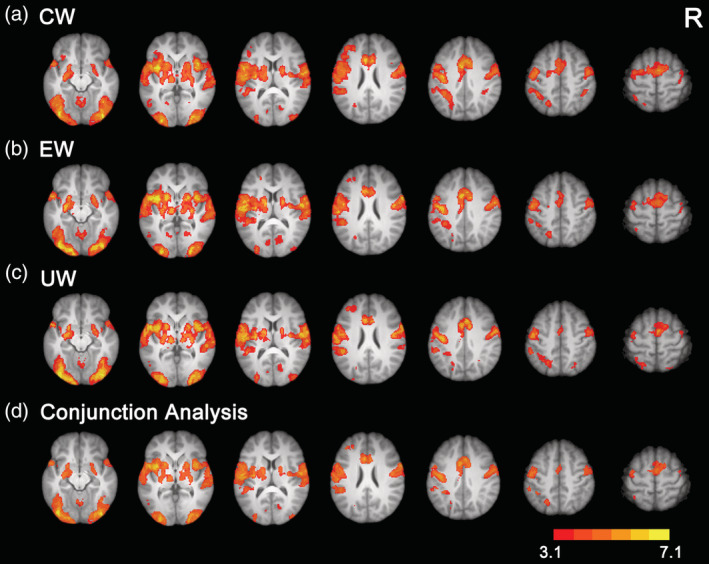
Brain activations for Chinese words (a), English words (b), and Uyghur words (c). Conjunction analysis showed that the three types of words elicited common activation in the typical reading network (d). All activations were thresholded at *Z* > 3.1 (whole‐brain corrected). CW, Chinese words; EW, English words; UW, Uyghur words; R, right

**FIGURE 3:**
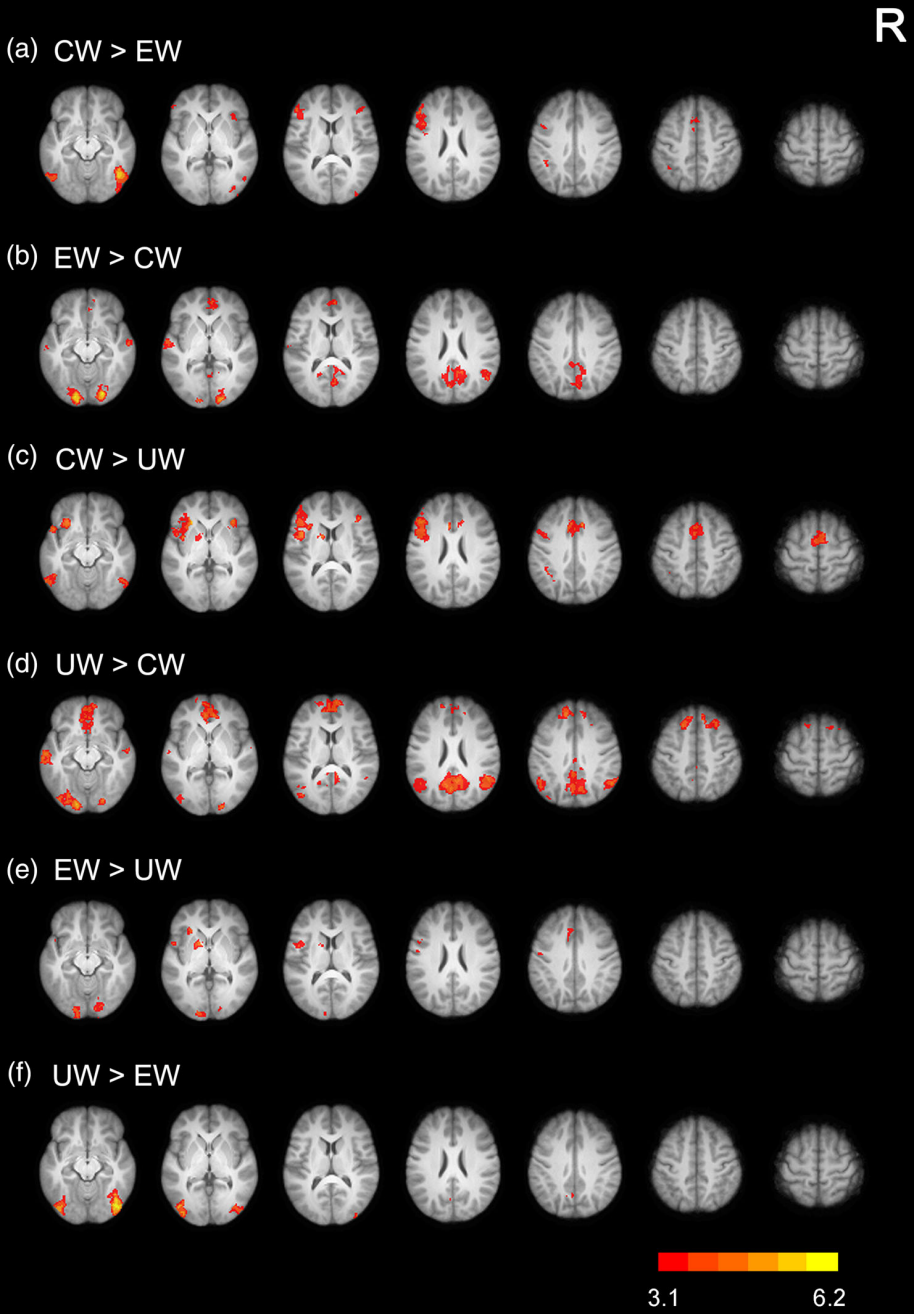
Brain regions showing differential neural activation across the three types of words (i.e., UW, CW, and EW). All activations were thresholded at *Z* > 3.1 (whole‐brain corrected). CW, Chinese words; EW, English words; UW, Uyghur words; R, right

Whole‐brain analysis was first used to investigate the neural activations of Uyghur, Chinese, and English words. We found that, the three types of words evoked common neural activation in the anterior cingulate cortex (ACC, extending to the supplementary motor cortex), bilateral prefrontal cortex, occipitoparietal cortex, and occipitotemporal cortex (see Figure 2 and Table S1). Direct comparisons between native and non‐native languages showed that Uyghur words evoked stronger activation in the bilateral occipital cortex and left precuneus cortex than English words and in the bilateral frontal pole, middle temporal gyrus, and AG than Chinese words. In contrast, Chinese and English words evoked stronger activation in the ACC, prefrontal cortex, and occipitotemporal cortex. Direct comparison between Chinese and English words revealed that Chinese words evoked stronger activation in the ACC, bilateral prefrontal cortex, occipitotemporal cortex, and left AG, whereas English words evoked stronger activation in the bilateral lateral temporal cortex, precuneus cortex, and occipital pole (see Figure 3 and Table S2).

### 3.3 Greater pattern similarity between Uyghur and English words than between Uyghur and Chinese words during word Reading

**FIGURE 4:**
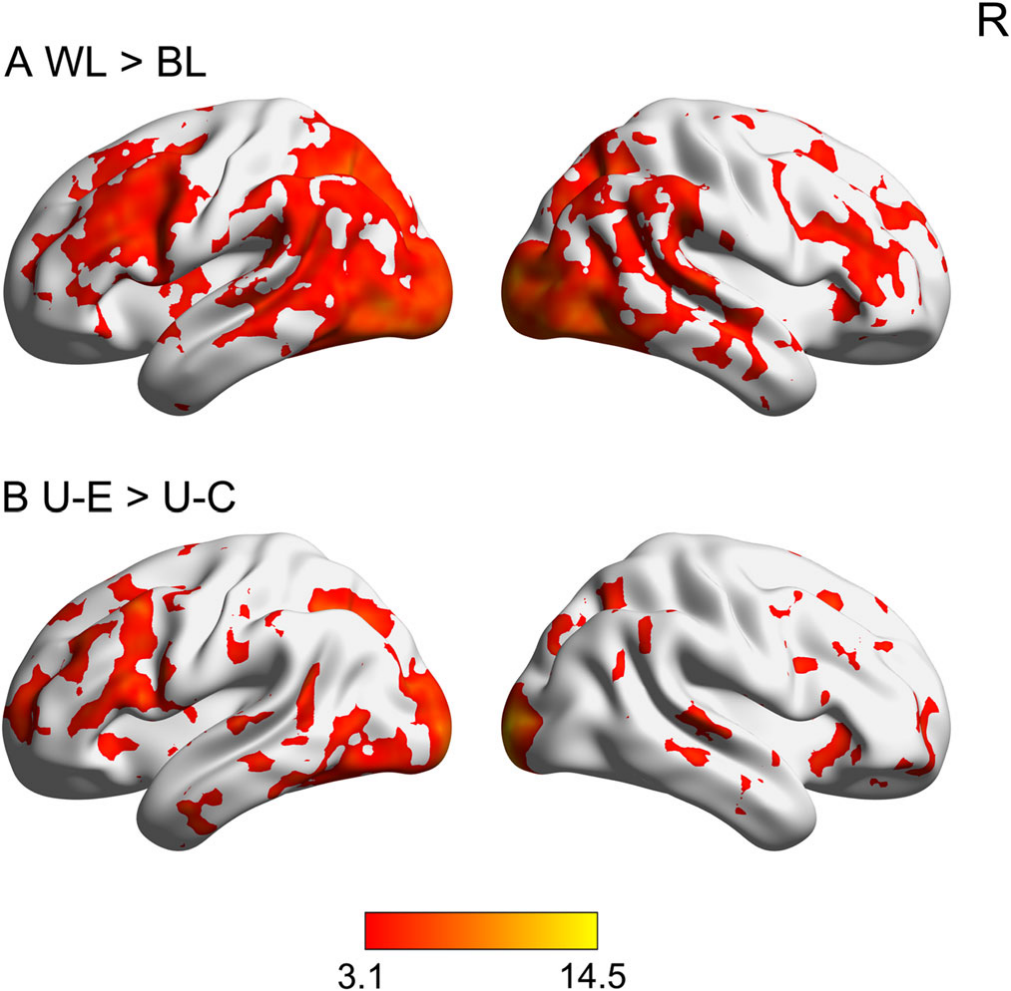
Brain maps for representational similarity analysis. The upper panel shows brain regions in which the within‐language pattern similarity (i.e., WL) was higher than the between‐language pattern similarity (i.e., BL) (a). The lower panel presents brain regions in which the pattern similarity between Uyghur and English (i.e., U‐E) was higher than that between Uyghur and Chinese (i.e., U‐C) (b). All activations were thresholded at Z > 3.1 (whole‐brain corrected). R, right

We first compared within‐language pattern similarity with between‐language pattern similarity in the two runs to investigate the validity of RSA in this research. We found that, compared with between‐language pattern similarity, within‐language pattern similarity was higher in the ACC, bilateral prefrontal cortex, temporoparietal cortex, and occipitotemporal cortex. No brain regions showed the reverse effect (see Figure 4a and Table S3). These results indicate that the RSA had good validity in this research.

We then verified the effects of language distance in orthographic transparency on cross‐language pattern similarity by comparing the pattern similarity between Uyghur and English words (U‐E) with that between Uyghur and Chinese words (U‐C). Because the language distance between Uyghur and Chinese words was larger than that between Uyghur and English words in terms of orthographic transparency, the pattern similarity of U‐E should be greater than that of U‐C in the neural network for word reading. Consistent with our expectation, whole‐brain RSA revealed greater pattern similarity for U‐E than that of U‐C in a wide neural network, especially in the left hemisphere, including the bilateral prefrontal cortex, lateral temporal cortex, occipital lobe, left SMG (extending to the superior parietal lobule), and precuneus cortex. In contrast, no brain regions showed greater pattern similarity for U‐C than for U‐E (see Figure 4b and Table S3). These results were confirmed after controlling for the effects of language proficiency and age of acquisition by adding the differences in reaction time and the age of acquisition between Chinese and English words as covariates in the second‐level analysis respectively (see Figures S1 and S4). These results indicate that language distance in orthographic transparency affects cross‐language pattern similarity between native and non‐native languages.

### 3.4 The effects of language distance on cross‐language pattern similarity in the brain regions for phonological processing

**FIGURE 5:**
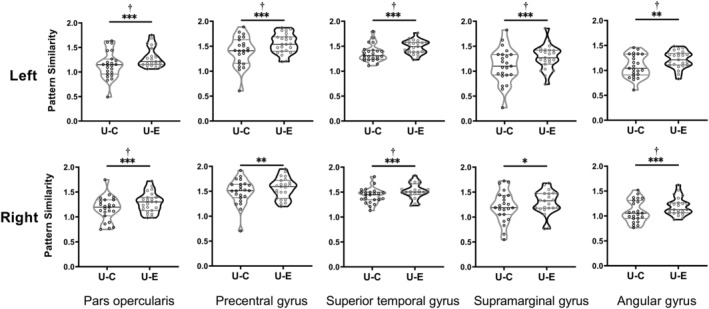
The effect of language distance on the cross‐language pattern similarity in the 10 predefined ROIs for word reading. Violin plots show the spread and differences in the pattern similarity between Uyghur and English words (U‐E) and that between Uyghur and Chinese words (U‐C). *p < .05, **p < .01, ***p < .001, ^†^ *p* < .005 (significance level after Bonferroni correction)

ROI‐based RSA was further performed to verify whether the effects of language distance in orthographic transparency occurred in brain areas responsible for phonological processing. In this analysis, we conducted one‐way repeated measures ANOVA to compare the pattern similarity of U‐C with that of U‐E within the 10 predefined ROIs for phonological processing. As shown in Figure 5 and Table S4, U‐E showed greater pattern similarity than U‐C in all ROIs, including in the bilateral PO, AG, pSTG, SMG, and PCG. Within those ROIs, eight ROIs, including the bilateral PO, pSTG, AG, left PCG, and SMG, survived to indicate significance after the Bonferroni correction (*p* < .005) (see Figure 5). These results suggest that a smaller language distance in orthographic transparency is related to greater cross‐language pattern similarity in brain areas for phonological processing, especially for regions in the left hemisphere.

To eliminate the potential confounding effect of language proficiency, we reran the above ANOVA in each ROI and included the differences in reaction time between Chinese and English words for all participants as a covariate. Greater pattern similarity for U‐E relative to that of U‐C was still found in seven ROIs, including the bilateral PO, pSTG, left PCG, SMG, and right AG (see Table S4). After Bonferroni correction, the bilateral pSTG and right PO remained significant. The effects of language distance in those three regions were still significant after controlling for the differences in reaction time between Uyghur and English words and those between Uyghur and Chinese words (the left pSTG: *F*(1,22) = 13.68, *p =* .001; the right pSTG: *F*(1,22) = 8.62, *p* = .008; and the right PO: *F*(1,22) = 9.13, *p* = .007). These results were also confirmed after controlling for the effects of the age of language acquisition (see Table S5). Therefore, these findings indicate that the differences in pattern similarity in the bilateral pSTG and right PO were not caused by language proficiency or the age of acquisition.

### 3.5 Brain regions showing the effect of language distance represented phonological information in Uyghur and English words

To directly explore the nature of the information represented in the eight ROIs showing significant language distance effect (i.e., the bilateral PO, pSTG, AG, left PCG, and SMG) (see Figure 5), we correlated the cross‐language neural dissimilarity matrix in those ROIs with three cross‐language predicted dissimilarity matrices (i.e., visual, phonological, and semantic prediction matrices) (see Figure S2). The results showed that the correlations between the neural dissimilarity matrix and phonological prediction matrix were significant in all eight ROIs for the paired U‐E (all *p*s *<* .01), but not in any ROIs for the paired U‐C (the smallest *p* = .085) (see Table 1). The lack of significant correlations for the paired U‐C was replicated even if the tonal information was excluded in the computation of the phonological prediction matrix (the smallest *p* = .052). These results were confirmed by the permutation test (see Figure S3). No regions showed significant correlations for the visual prediction matrix or the semantic prediction matrix. The significant correlations between the neural dissimilarity matrix and phonological prediction matrix were also confirmed after controlling for the visual and semantic prediction matrices (see Table 1). These results indicate that greater pattern similarity between Uyghur and English in brain regions for phonological processing can be accounted for their common activation of phonological information. In addition to the brain regions for phonological processing, other brain regions for word processing, such as the bilateral middle temporal gyrus, left pars triangularis, inferior temporal gyrus, fusiform gyrus, and superior parietal lobule, also showed significant language distance effects, these effects were driven by a common activation of phonological information in Uyghur and English (see Tables S7 and S8).

**TABLE 1:** Spearman correlations between cross‐language neural dissimilarity matrices and the three prediction matrices in the eight ROIs for the two cross‐language pairs

Brain regions	Visual		Phonological		Semantic		Phonological (adjusted)	
	*r*	*p*	*r*	*p*	*r*	*p*	*r*	*p*
*Uyghur‐English*								
Left pars opercularis	.005	.359	.010	.004**	.000	.971	.009	.004**
Right pars opercularis	.008	.118	.014	.000***	.001	.683	.013	.000***
Left precentral gyrus	.000	.958	.014	.000***	.002	.542	.014	.001**
Left superior temporal gyrus	.005	.393	.012	.000***	.005	.230	.012	.000***
Right superior temporal gyrus	.009	.078	.016	.000***	.004	.351	.016	.000***
Left supramarginal gyrus	.002	.764	.011	.002**	.005	.167	.011	.002**
Left angular gyrus	.002	.745	.014	.000***	.004	.490	.014	.000***
Right angular gyrus	.008	.089	.012	.001**	.006	.161	.012	.001**
*Uyghur‐Chinese*								
Left pars opercularis	.001	.944	.001	.707	.001	.588	.001	.706
Right pars opercularis	.007	.308	.006	.085	.001	.845	.006	.086
Left precentral gyrus	.010	.128	.004	.115	−.001	.717	.004	.119
Left superior temporal gyrus	.014	.076	.003	.358	.000	.867	.003	.371
Right superior temporal gyrus	.009	.225	.005	.143	.002	.506	.005	.146
Left supramarginal gyrus	.007	.417	.002	.597	−.003	.331	.002	.610
Left angular gyrus	.007	.475	.001	.816	.001	.713	.001	.820
Right angular gyrus	.006	.362	−.002	.589	−.002	.461	−.002	.580

*Note:* Phonological (adjusted) represents partial correlation between neural dissimilarity matrix and phonological prediction matrix after controlling for visual and semantic prediction matrices.

^*^ *p* < .05.

^**^ *p* < .01.

^***^ *p* < .001.

## 4 DISCUSSION

In this study, we used RSA to quantify how language distance in orthographic transparency affected the similarity between the neural patterns evoked when Uyghur‐Chinese‐English trilinguals read words in their native and non‐native languages. Behavioral results showed that, consistent with previous studies (Hamada & Koda, 2008; Pae, Sun, Mano, & Kwon, 2016), the degree of similarity in orthography between native and non‐native languages affected the naming speed of words in non‐native languages. Specifically, subjects responded more quickly to words in a non‐native language with more similar orthography to their native language. Imaging data showed that reading words in the three languages generally elicited common activations in brain regions for word reading, including the bilateral prefrontal cortex, occipitoparietal cortex and occipitotemporal cortex (Cao et al., 2013; Kim et al., 2016; Kim et al., 2017; Nakada et al., 2001; Tan et al., 2003; van de Putte et al., 2017). Consistent with previous studies (Mei et al., 2014; Mei, Xue, Lu, He, et al., 2015; Paulesu et al., 2000), reading an opaque language (i.e., Chinese) elicited stronger activations in the inferior frontal gyrus and occipitotemporal cortex for lexical processing than reading a transparent language (i.e., Uyghur). More importantly, RSA revealed greater cross‐language pattern similarity within brain areas responsible for phonological processing for language pairs (i.e., Uyghur and English) with a small language distance relative to those with a large language distance (i.e., Uyghur and Chinese) in terms of orthographic transparency. These results suggest that language distance in orthographic transparency affects pattern similarity between native and non‐native languages.

As discussed in the Introduction, orthographic transparency has a great impact on the cognitive and neural mechanisms of phonological access (Bigozzi et al., 2016; Buetler et al., 2014; Cao et al., 2017; Coltheart et al., 2001; Meschyan & Hernandez, 2006; Nosarti et al., 2010; Simon et al., 2006). Thus, language distance in orthographic transparency may modulate the activation similarity between native and non‐native languages. Consistent with this view, one recent study found that native and non‐native languages with similar orthography showed greater overlap in activation than those with dissimilar orthography (Kim et al., 2016). Based on these findings, our study further precisely estimated the neural pattern similarity between native and non‐native languages by using RSA (Li et al., 2019) and specified the brain areas showing the effects of language distance in orthographic transparency. The results showed that greater cross‐language pattern similarity was associated with language pairs with a smaller language distance in orthographic transparency in a number of brain regions for phonological processing, including the bilateral PO, pSTG, left PCG, SMG, and AG. These results provide direct neuroimaging evidence for the influence of language distance in orthographic transparency on cross‐language pattern similarity.

Greater pattern similarity of U‐E relative to U‐C in brain regions for phonological processing can be attributed to the similarity in the involvement of phonological access pathways between native and non‐native languages. Specifically, native and non‐native languages with smaller distances in orthographic transparency show greater cross‐language pattern similarity because of their similar engagement in phonological access pathways. Consistent with this view, previous behavioral studies have found that native and non‐native languages with similar orthographic transparency adopt common orthographic processing skills in phonological access (Abu‐Rabia & Shakkour, 2014; Kahn‐Horwitz, Shimron, & Sparks, 2005). This view is also supported by our results that subjects responded faster to words in a non‐native language with more similar orthography to their native language in the word naming task. In addition, previous neuroimaging studies have revealed that reading in more transparent orthography depends more on the orthography‐to‐phonology mapping pathway and consequently shows more activation in brain regions for phonological processing (e.g., the PCG, dorsal inferior frontal gyrus, and temporoparietal cortex) (Cao et al., 2017; Cattinelli, Borghese, Gallucci, & Paulesu, 2013; Jobard et al., 2003; Mechelli et al., 2005; Mei, Xue, Lu, Chen, et al., 2015; Tan et al., 2005). Thus, compared with Chinese, Uyghur and English reading are more likely to recruit the orthography‐to‐phonology mapping pathway and consequently show greater cross‐language pattern similarity within brain regions for phonological processing. Although Chinese is different from Uyghur and English in tonality in addition to orthography, we believe that the language distance effects in this study reflected the differences in orthographic transparency but not the differences in tonality, because the correlations between neural dissimilarity matrices and the phonological prediction matrix for the paired U‐C were not significant regardless of whether the tonal information was included.

The common engagement of the orthography‐to‐phonology mapping pathway in Uyghur and English reading was confirmed by the RSA on the cross‐language neural dissimilarity matrix and three predicted dissimilarity matrices. Specifically, the neural pattern signal was found to be associated with phonological information in the paired U‐E but not with visual or semantic information. Therefore, greater pattern similarity between Uyghur and English reflects their similar mechanisms of phonological processing in word reading. Our results of phonological representation in the eight ROIs are also consistent with previous findings that the left temporoparietal cortex (e.g., SMG, AG, and pSTG) is responsible for the GPC (Booth et al., 2006; Fiez et al., 1996; Howard et al., 1992; Price, 2012; Tan et al., 2005; Warburton et al., 1996) and that the dorsal inferior frontal gyrus and PCG play important roles in syllabification and articulation in speech production (Fedorenko & Blank, 2020; Long et al., 2016; Mei, Xue, Lu, He, et al., 2015; Niu et al., 2016; Price, 2012).

It is worth noting that the brain regions showing the effects of language distance in orthographic transparency were mainly located in the left hemisphere. These findings are in accordance with the traditional view of the superiority of the left hemisphere in language processing (Balsamo et al., 2002; Josse, Mazoyer, Crivello, & Tzourio‐Mazoyer, 2003; Josse & Tzourio‐Mazoyer, 2004; Wilenius, Lehtinen, Paetau, Salmelin, & Kirveskari, 2018). Consistently, previous neuroimaging research has revealed left‐lateralized activations in brain areas for language processing during word reading, especially for words in alphabetic scripts (Cohen et al., 2002; Mei, Xue, Lu, Chen, et al., 2015; Nelson et al., 2009; Vigneau, Jobard, Mazoyer, & Tzourio‐Mazoyer, 2005).

Our results of the language distance effects have important implications for our understanding of cross‐language influences. Our study suggests that when native and non‐native languages have similar orthographic transparency, the brain network involved in native language processing is effectively reutilized during non‐native language learning (Kim et al., 2016). Such reutilization has been found to improve behavioral performance in non‐native language learning (Hamada & Koda, 2008; Pae et al., 2016). In contrast, when learning a non‐native language in which the orthographic transparency differs from that of the native language, different neural computations are involved (Mei, Xue, Lu, He, et al., 2015), which increases the difficulty of learning that non‐native language. Future research should identify the critical brain regions or neural networks involved (or that should be involved) in foreign language learning when the native and non‐native languages do not have the same type of orthographic transparency. Such knowledge can help to develop or improve existing clinical and educational interventions for foreign language learning (Cohen Kadosh, Soskic, Iuculano, Kanai, & Walsh, 2010; Meinzer et al., 2014; Xue et al., 2017). It is also possible to identify brain regions that should have been, but are typically not, involved in learning a new language with a different type of orthographic transparency. For example, it has been revealed that the left SMG is not sufficiently active when Chinese speakers read in English (Mei, Xue, Lu, He, et al., 2015). Therefore, Xue et al. (2017) applied the anodal tDCS to the left SMG, which was able to facilitate the acquisition of lexical learning in an alphabetic language in Chinese speakers (Xue et al., 2017).

There are three limitations in the present research. First, the two non‐native languages differed in language proficiency (i.e., Chinese and English words), which may influence the pattern similarity between native and non‐native languages (Cao, 2015; Cao et al., 2013; Gao et al., 2017; Li et al., 2019; Stein et al., 2009). Although we used the differences in reaction time between Chinese and English words as a covariate in the analysis, the potential effect of language proficiency might not be completely ruled out. Therefore, future studies should confirm our results by using two groups of bilinguals who have comparable language proficiency in non‐native languages. Second, the two non‐native languages differed in visual appearance, which may also have confounded the effects of language distance in orthographic transparency on cross‐language pattern similarity. Consistent with this view, we found the effects of language distance in orthographic transparency in the visual cortex (i.e., bilateral occipital cortex). Thus, future research study should use a strictly controlled artificial language training paradigm to control for the confounding effects of visual appearance. Finally, due to the large noise in the scanner, we collected the behavioral data of the naming task after scanning. Although the major conclusions in this study were drawn based on the imaging data, repeated naming of experimental materials after scanning may have affected our behavioral results due to the practice effect. Future studies should test the effects of language distance on cross‐language pattern similarity by recording subjects' oral responses during scanning.

## 5 CONCLUSION

In conclusion, using Uyghur‐Chinese‐English trilinguals and RSA, this study revealed that greater cross‐language pattern similarity was associated with a smaller language distance in orthographic transparency in brain areas for phonological processing, especially in the left hemisphere. Further analysis confirmed that those brain regions represented phonological information. These results provide direct neuroimaging evidence for the modulatory effect of language distance in orthographic transparency on cross‐language pattern similarity in the brain regions for phonological processing.

## CONFLICT OF INTERESTS

The authors declare no conflict of interest.

## AUTHOR CONTRIBUTIONS

**Jie Dong** and **Leilei Mei**: Designed research. **Jie Dong**, **Aqian Li**, **Jing Qu**, **Nan Jiang**, **Yue Sun**, and **Liyuan Hu**: Performed research. **Jie Dong** and **Leilei Mei**: Analyzed the data. **Jie Dong**, **Aqian Li**, **Chuansheng Chen**, **Jing Qu**, **Nan Jiang**, and **Leilei Mei**: Wrote and approved the article.

## Supporting information


**TABLE S1** **Brain regions showing activations for Chinese words (CW), English words (EW), and Uyghur words (UW).**
**TABLE S2 Brain regions showing different activations for Chinese words (CW), English words (EW) and Uyghur words (UW).**
**TABLE S3 Brain regions for the whole‐brain representational similarity analysis.**
**TABLE S4 The comparisons of cross‐language pattern similarity in the 10 predefined ROIs after controlling for language profiency.**
**TABLE S5 The comparisons of cross‐language pattern similarity in the 10 predefined ROIs after controlling for age of acquisition.**
**TABLE S6 Stimuli of the three languages used in this study.**
**TABLE S7 The comparisons of cross‐language pattern similarity in the 22 brain regions for comprehensive word processing.**
**TABLE S8 Spearman correlations between cross‐language neural dissimilarity matrices and the three prediction matrices in the 13 brain regions showing significant effects in Table S7.**
**FIGURE S1.** Brain maps for representational similarity analysis after controlling for the differences in reaction time between Chinese and English words. It presents brain regions showing greater pattern similarity between Uyghur and English than that between Uyghur and Chinese. All activations were thresholded at Z > 3.1 (whole‐brain corrected). R = right.
**FIGURE S2.** The three prediction matrices for each cross‐language pair (i.e., Uyghur‐English and Uyghur‐Chinese). For the visual prediction matrices (A), a binary silhouette of each word was used to compute the pixel‐wise nonoverlap regions of the two images in each cross‐language pair. For the phonological prediction matrices (B), we used the second coding scheme from the MatchCalculator tool, which was developed by Colin Davis (www.pc.rhul.ac.uk/staff/c.davis/Utilities/MatchCalc/). It was calculated as 1 minus the proportion of same‐position phonemes shared across the two words in each cross‐language pair. The semantic prediction matrices (C) were estimated by dividing the words in the three languages into twelve categories according to their semantic similarity. Item pairs from the same semantic category were denoted as 0, and pairs from different categories were denoted as 1.
**FIGURE S3**. The histogram plots of permutation test in the 8 ROIs which showed significant correlations between cross‐language neural dissimilarity matrix and phonological prediction matrix. The green line indicates the actual correlation between neural dissimilarity matrix and phonological prediction matrix for all Uyghur‐English item pairs, and the red line indicates the fifth percentile (0.05) of the distribution. X‐axis represents the correlation coefficients.
**FIGURE S4**. Brain maps for representational similarity analysis after controlling for the differences in age of acquisition between Chinese and English. It presents brain regions showing greater pattern similarity between Uyghur and English than that between Uyghur and Chinese. All activations were thresholded at Z > 3.1 (whole‐brain corrected). R = right.

## Data Availability

Data are available upon request.
