# Supplementary material for: Language distance in orthographic transparency affects cross‐language pattern similarity between native and non‐native languages
Source: Hum Brain Mapp. 2020 Oct 28;42(4):893–907. doi: 10.1002/hbm.25266 (PMC7856648; doi:10.1002/hbm.25266)
Supplement: Supplementary file 1 — TABLE S1 Brain regions showing activations for Chinese words (CW), English words (EW), and Uyghur words (UW). TABLE S2 Brain regions showing different activations for Chinese words (CW), English words (EW) and Uyghur words (UW). TABLE S3 Brain regions for the whole‐brain representational similarity analysis. TABLE S4 The comparisons of cross‐language pattern similarity in the 10 predefined ROIs after controlling for language profiency. TABLE S5 The comparisons of cross‐language pattern similarity in the 10 predefined ROIs after controlling for age of acquisition. TABLE S6 Stimuli of the three languages used in this study. TABLE S7 The comparisons of cross‐language pattern similarity in the 22 brain regions for comprehensive word processing. TABLE S8 Spearman correlations between cross‐language neural dissimilarity matrices and the three prediction matrices in the 13 brain regions showing significant effects in Table S7. FIGURE S1. Brain maps for representational similarity analysis after controlling for the differences in reaction time between Chinese and English words. It presents brain regions showing greater pattern similarity between Uyghur and English than that between Uyghur and Chinese. All activations were thresholded at Z > 3.1 (whole‐brain corrected). R = right. FIGURE S2. The three prediction matrices for each cross‐language pair (i.e., Uyghur‐English and Uyghur‐Chinese). For the visual prediction matrices (A), a binary silhouette of each word was used to compute the pixel‐wise nonoverlap regions of the two images in each cross‐language pair. For the phonological prediction matrices (B), we used the second coding scheme from the MatchCalculator tool, which was developed by Colin Davis (www.pc.rhul.ac.uk/staff/c.davis/Utilities/MatchCalc/). It was calculated as 1 minus the proportion of same‐position phonemes shared across the two words in each cross‐language pair. The semantic prediction matrices (C) were estimated by dividing the words in the three langu [file HBM-42-893-s001.doc]

Supplementary Information

**Language Distance in Orthographic Transparency Affects Cross-Language Pattern Similarity Between Native and Non-native Languages**

**Jie Dong^1,2,3,4^, Aqian Li^1,2,3,4^, Chuansheng Chen^5^, Jing Qu^1,2,3,4^, Nan Jiang^1,2,3,4^, Yue Sun^1,2,3,4^, Liyuan Hu^1,2,3,4^, Leilei Mei^1,2,3,4^**^*^

^1^ Key Laboratory of Brain, Cognition and Education Sciences (South China Normal University), Ministry of Education

^2^ School of Psychology, South China Normal University, 510631 Guangzhou, China

^3^ Center for Studies of Psychological Application, South China Normal University, 510631 Guangzhou, China

^4^ Guangdong Key Laboratory of Mental Health and Cognitive Science, South China Normal University, 510631 Guangzhou, China

^5^ Department of Psychological Science, University of California, Irvine, California, USA

**^*^Correspondence**

Leilei Mei, School of Psychology, South China Normal University, Guangzhou, 510631, China

E-mail: [mll830925@126.com](mailto:mll830925@126.com)

**This PDF file includes:**

Tables S1 to S8

Figures S1 to S4

**Table S1 Brain regions showing activations for Chinese words (CW), English words (EW), and Uyghur words (UW).**

| **Brain regions** | Left | | | | |  |  | Right | | | |
| --- | --- | --- | --- | --- | --- | --- | --- | --- | --- | --- | --- |
|  | Voxels | x | y | z | Z |  | Voxels | x | y | z | Z |
| **CW** |  |  |  |  |  |  |  |  |  |  |  |
| Anterior cingulate gyrus/ supplementary motor cortex | 1325 | -4 | 20 | 26 | 5.84 |  | 1267 | 6 | 18 | 26 | 6.09 |
| Temporal pole/ inferior frontal gyrus | 2364 | -52 | 12 | -8 | 5.64 |  | 1593 | 56 | 18 | -8 | 4.96 |
| Precentral gyrus | 3306 | -38 | 6 | -4 | 6.18 |  | 2352 | 46 | 6 | -4 | 6.38 |
| Superior temporal gyrus | 1966 | -62 | -22 | 0 | 4.64 |  | 1475 | 70 | -22 | 0 | 4.65 |
| Supramarginal gyrus/ superior parietal lobule/ angular gyrus | 233 | -42 | -42 | 40 | 5.21 |  |  |  |  |  |  |
| Lateral occipital cortex/ fusiform gyrus | 6999 | -32 | -88 | 2 | 5.63 |  | 6869 | 40 | -86 | -12 | 6.45 |
|  |  |  |  |  |  |  |  |  |  |  |  |
| **EW** |  |  |  |  |  |  |  |  |  |  |  |
| Anterior cingulate gyrus/ supplementary motor cortex | 140 | -6 | 16 | 28 | 6.64 |  | 1215 | 2 | 14 | 28 | 5.53 |
| Temporal pole/ inferior frontal gyrus | 2350 | -52 | 12 | -8 | 5.29 |  | 2361 | 56 | 10 | -6 | 6.00 |
| Insular cortex/ precentral gyrus | 1160 | -42 | 12 | -4 | 6.60 |  | 1181 | 34 | 6 | 4 | 5.50 |
| Superior temporal gyrus | 1130 | -62 | -8 | 2 | 5.29 |  | 1221 | 56 | -28 | 2 | 5.24 |
| Lingual gyrus/ precuneus cortex | 911 | -12 | -64 | 2 | 3.37 |  | 187 | 18 | -60 | 2 | 3.65 |
| Lateral occipital cortex/ fusiform gyrus | 6121 | -40 | -84 | -10 | 6.64 |  | 226 | 24 | -90 | -4 | 5.25 |
|  |  |  |  |  |  |  |  |  |  |  |  |
| **UW** |  |  |  |  |  |  |  |  |  |  |  |
| Frontal pole | 1198 | -28 | 44 | 24 | 3.70 |  |  |  |  |  |  |
| Anterior cingulate gyrus/ supplementary motor cortex | 783 | -6 | 16 | 28 | 5.99 |  |  |  |  |  |  |
| Temporal pole/ inferior frontal gyrus | 888 | -54 | 10 | -8 | 5.96 |  | 4677 | 54 | 10 | -6 | 6.68 |
| Superior temporal gyrus/ middle temporal gyrus | 534 | -60 | -6 | 0 | 6.04 |  | 4194 | 68 | -18 | 6 | 4.90 |
| Precentral gyrus | 1136 | -40 | -14 | 36 | 6.04 |  | 3813 | 56 | -4 | 34 | 5.04 |
| Supramarginal gyrus/ angular gyrus | 232 | -50 | -42 | 22 | 4.99 |  |  |  |  |  |  |
| Superior parietal lobule | 235 | -38 | -52 | 54 | 4.77 |  |  |  |  |  |  |
| Fusiform gyrus | 1147 | -40 | -68 | -18 | 6.17 |  | 182 | 44 | -60 | -16 | 6.02 |
| Lateral occipital cortex/ precuneus cortex | 6476 | -40 | -82 | -12 | 7.00 |  | 130 | 26 | -58 | 42 | 3.94 |

**Table S2 Brain regions showing different activations** **for Chinese words (CW), English words (EW) and Uyghur words (UW).**

| **Brain regions** | Left | | | | |  | Right | | | | |
| --- | --- | --- | --- | --- | --- | --- | --- | --- | --- | --- | --- |
|  | Voxels | x | y | z | Z |  | Voxels | x | y | z | Z |
| **CW > EW** |  |  |  |  |  |  |  |  |  |  |  |
| Supplementary motor cortex/ anterior cingulate gyrus | 222 | -4 | 4 | 50 | 3.61 |  |  |  |  |  |  |
| Inferior frontal gyrus/ middle frontal gyrus | 835 | -42 | 24 | 18 | 4.20 |  | 193 | 40 | 28 | 6 | 3.69 |
| Supramarginal gyrus/ angular gyrus | 153 | -44 | -42 | 38 | 4.01 |  |  |  |  |  |  |
| Inferior temporal gyrus | 130 | -62 | -58 | -18 | 3.35 |  |  |  |  |  |  |
| Lateral occipital cortex | 182 | -60 | -64 | -12 | 4.37 |  | 819 | 48 | -60 | -12 | 5.28 |
|  |  |  |  |  |  |  |  |  |  |  |  |
| **EW > CW** |  |  |  |  |  |  |  |  |  |  |  |
| Paracingulate gyrus/ frontal pole |  |  |  |  |  |  | 485 | 4 | 50 | 4 | 4.67 |
| Superior temporal gyrus/ middle temporal gyrus | 262 | -64 | -8 | -2 | 4.03 |  | 183 | 60 | -10 | -10 | 4.59 |
| Precuneus cortex | 213 | -6 | -68 | 28 | 4.05 |  | 1412 | 10 | -66 | 20 | 3.77 |
| Fusiform gyrus/ occipital pole | 831 | -18 | -90 | -10 | 6.00 |  | 706 | 22 | -88 | -8 | 6.12 |
|  |  |  |  |  |  |  |  |  |  |  |  |
| **CW > UW** |  |  |  |  |  |  |  |  |  |  |  |
| Anterior cingulate gyrus/ supplementary motor cortex | 843 | -6 | 20 | 26 | 4.86 |  | 273 | 6 | 8 | 58 | 4.44 |
| Temporal pole/ inferior frontal gyrus | 1518 | -52 | 18 | -14 | 4.52 |  | 196 | 34 | 28 | 2 | 4.32 |
| Inferior temporal gyrus/ lateral occipital cortex | 378 | -62 | -58 | -18 | 4.74 |  | 295 | 56 | -62 | -16 | 4.11 |
|  |  |  |  |  |  |  |  |  |  |  |  |
| **UW > CW** |  |  |  |  |  |  |  |  |  |  |  |
| Frontal pole | 670 | -14 | 40 | 40 | 4.22 |  | 2531 | 12 | 40 | 40 | 3.87 |
| Middle temporal gyrus | 411 | -62 | -14 | -12 | 4.34 |  | 225 | 54 | -6 | -24 | 4.19 |
| Angular gyrus | 152 | -52 | -56 | 28 | 4.16 |  | 156 | 52 | -50 | 24 | 4.41 |
| Precuneus cortex | 1513 | -4 | -68 | 30 | 5.06 |  | 623 | 6 | -60 | 28 | 5.09 |
| Fusiform gyrus/ lateral occipital cortex | 182 | -16 | -90 | -10 | 5.70 |  |  |  |  |  |  |
|  |  |  |  |  |  |  |  |  |  |  |  |
| **EW > UW** |  |  |  |  |  |  |  |  |  |  |  |
| Anterior cingulate gyrus | 124 | -10 | 16 | 34 | 3.53 |  |  |  |  |  |  |
| Inferior frontal gyrus/ temporal pole | 180 | -48 | 8 | 10 | 3.56 |  |  |  |  |  |  |
| Precentral gyrus | 189 | -52 | 6 | 6 | 4.33 |  |  |  |  |  |  |
| Occipital pole | 448 | -14 | -96 | -8 | 4.92 |  | 371 | 14 | -92 | -8 | 4.46 |
|  |  |  |  |  |  |  |  |  |  |  |  |
| **UW > EW** |  |  |  |  |  |  |  |  |  |  |  |
| Precuneus cortex | 117 | -4 | -68 | 30 | 3.60 |  |  |  |  |  |  |
| Lateral occipital cortex | 927 | -42 | -76 | -4 | 4.96 |  | 1001 | 44 | -78 | -12 | 6.25 |

**Table S3** **Brain regions for the whole-brain representational similarity analysis.**

| **Brain regions** | Left | | | | |  | Right | | | | |
| --- | --- | --- | --- | --- | --- | --- | --- | --- | --- | --- | --- |
|  | Voxels | x | y | z | Z |  | Voxels | x | y | z | Z |
| **WL > BL** |  |  |  |  |  |  |  |  |  |  |  |
| Frontal pole | 1425 | -26 | 52 | 8 | 4.05 |  | 2074 | 48 | 38 | 10 | 4.36 |
| Inferior frontal gyrus | 1074 | -44 | 34 | 16 | 6.45 |  | 557 | 46 | 34 | 12 | 4.09 |
| Anterior cingulate gyrus/ supplementary motor cortex | 442 | -6 | 14 | 28 | 4.37 |  | 357 | 4 | 20 | 28 | 3.80 |
| Precentral gyrus | 1325 | -56 | 6 | 34 | 6.59 |  | 668 | 56 | 8 | 8 | 3.22 |
| Middle temporal gyrus/ superior temporal gyrus | 613 | -54 | -18 | -16 | 6.77 |  | 826 | 56 | -18 | -16 | 4.85 |
| Supramarginal gyrus/ superior parietal lobule | 885 | -56 | -32 | 36 | 6.64 |  | 879 | 56 | -30 | 44 | 4.52 |
| Fusiform gyrus | 1439 | -24 | -86 | -6 | 9.16 |  | 1376 | 32 | -80 | -16 | 5.90 |
| Occipital pole | 1961 | -20 | -98 | -4 | 11.63 |  | 1741 | 20 | -98 | -2 | 5.71 |
|  |  |  |  |  |  |  |  |  |  |  |  |
| **BL > WL** |  |  |  |  |  |  |  |  |  |  |  |
| None |  |  |  |  |  |  |  |  |  |  |  |
|  |  |  |  |  |  |  |  |  |  |  |  |
| **U-E > U-C** |  |  |  |  |  |  |  |  |  |  |  |
| Frontal pole/ frontal orbital cortex | 2091 | -52 | 36 | -12 | 4.74 |  | 1246 | 8 | 54 | 46 | 4.22 |
| Inferior frontal gyrus | 924 | -42 | 10 | 22 | 6.60 |  | 332 | 48 | 16 | 8 | 3.18 |
| Superior temporal gyrus/ precentral gyrus | 409 | -62 | -28 | -6 | 6.71 |  | 461 | 62 | -22 | 4 | 6.79 |
| Middle temporal gyrus | 491 | -58 | -46 | 4 | 3.68 |  | 384 | 58 | -40 | 4 | 3.50 |
| Supramarginal gyrus/ angular gyrus/ superior parietal lobule | 226 | -52 | -48 | 16 | 4.90 |  |  |  |  |  |  |
| Precuneus cortex | 1259 | -4 | -62 | 46 | 4.34 |  |  |  |  |  |  |
| Fusiform gyrus | 1133 | -20 | -88 | -8 | 6.14 |  | 665 | 26 | -86 | -4 | 5.08 |
| Occipital pole | 1812 | -18 | -98 | -4 | 14.17 |  | 1363 | 18 | -94 | -10 | 14.15 |
|  |  |  |  |  |  |  |  |  |  |  |  |
| **U-C> U-E** |  |  |  |  |  |  |  |  |  |  |  |
| None |  |  |  |  |  |  |  |  |  |  |  |
|  |  |  |  |  |  |  |  |  |  |  |  |

Notes: WL = pattern similarity within languages.

BL = pattern similarity between languages.

U-C = pattern similarity between Uyghur and Chinese words.

U-E = pattern similarity between Uyghur and English words.

**Table S4 The comparisons of cross-****language pattern similarity in the 10 predefined ROIs after controlling for language profiency.**

| **ROIs** | **U-C** | **U-E** | **F^a^** | **P^a^** | **F^b^** | **P^b^** | **F^c^** | **P^c^** |
| --- | --- | --- | --- | --- | --- | --- | --- | --- |
|  | **M** | **M** |  |  |  |  |  |  |
| **Left** |  |  |  |  |  |  |  |  |
| Pars opercularis | 1.14 | 1.30 | 17.98 | 0.000***^†^ | 9.40 | 0.006** | 7.33 | 0.014* |
| Precentral gyrus | 1.40 | 1.55 | 17.14 | 0.000***^†^ | 6.26 | 0.021* | 4.19 | 0.054 |
| Superior temporal gyrus | 1.36 | 1.48 | 40.00 | 0.000***^†^ | 23.16 | 0.000***^†^ | 13.68 | 0.001**^†^ |
| Supramarginal gyrus | 1.11 | 1.28 | 16.66 | 0.000***^†^ | 8.14 | 0.010* | 2.54 | 0.127 |
| Angular gyrus | 1.09 | 1.22 | 12.05 | 0.002**^†^ | 3.60 | 0.072 | 2.62 | 0.121 |
| **Right** |  |  |  |  |  |  |  |  |
| Pars opercularis | 1.15 | 1.30 | 29.48 | 0.000***^†^ | 15.71 | 0.001**^†^ | 9.13 | 0.007** |
| Precentral gyrus | 1.48 | 1.58 | 8.78 | 0.007** | 2.24 | 0.150 | 2.07 | 0.165 |
| Superior temporal gyrus | 1.43 | 1.53 | 24.74 | 0.000***^†^ | 13.05 | 0.002**^†^ | 8.62 | 0.008** |
| Supramarginal gyrus | 1.18 | 1.30 | 5.30 | 0.031* | 0.64 | 0.433 | 0.44 | 0.515 |
| Angular gyrus | 1.07 | 1.19 | 18.40 | 0.000***^†^ | 7.72 | 0.011* | 2.09 | 0.164 |

Notes: U-C = pattern similarity between Uyghur and Chinese words.

U-E = pattern similarity between Uyghur and English words.

**^a^**The comparisons of pattern similarity between U-C and U-E.

**^b^**The comparisons of pattern similarity between U-C and U-E after controlling for covariates (i.e., the reaction time difference between Chinese and English words).

**^c^**The comparisons of pattern similarity between U-C and U-E while controlling for covariates (i.e., the reaction time difference between Uyghur and Chinese words, Uyghur and English words).

**p* < 0.05, ***p* < 0.01 and ****p* < 0.001, ^†^*p* < 0.005 (significance level after Bonferroni correction).

**Table S5 The comparisons of cross-language pattern similarity in the 10 predefined ROIs after controlling for age of acquisition.**

| **ROIs** | **U-C** | **U-E** | **F^a^** | **P^a^** | **F^b^** | **P^b^** | **F^c^** | **P^c^** |
| --- | --- | --- | --- | --- | --- | --- | --- | --- |
|  | **M** | **M** |  |  |  |  |  |  |
| **Left** |  |  |  |  |  |  |  |  |
| Pars opercularis | 1.14 | 1.30 | 17.98 | 0.000***† | 15.06 | 0.001**† | 14.39 | 0.001**† |
| Precentral gyrus | 1.40 | 1.55 | 17.14 | 0.000***† | 14.75 | 0.001**† | 14.20 | 0.001**† |
| Superior temporal gyrus | 1.36 | 1.48 | 40.00 | 0.000***† | 35.09 | 0.000***† | 32.95 | 0.000***† |
| Supramarginal gyrus | 1.11 | 1.28 | 16.66 | 0.000***† | 14.36 | 0.001**† | 13.58 | 0.001**† |
| Angular gyrus | 1.09 | 1.22 | 12.05 | 0.002**† | 9.85 | 0.005** | 9.46 | 0.006** |
| **Right** |  |  |  |  |  |  |  |  |
| Pars opercularis | 1.15 | 1.30 | 29.48 | 0.000***^†^ | 25.75 | 0.000***^†^ | 28.48 | 0.000***^†^ |
| Precentral gyrus | 1.48 | 1.58 | 8.78 | 0.007** | 7.20 | 0.014* | 6.71 | 0.017* |
| Superior temporal gyrus | 1.43 | 1.53 | 24.74 | 0.000***^†^ | 22.19 | 0.000***^†^ | 23.58 | 0.000***^†^ |
| Supramarginal gyrus | 1.18 | 1.30 | 5.30 | 0.031* | 3.94 | 0.060 | 3.60 | 0.072 |
| Angular gyrus | 1.07 | 1.19 | 18.40 | 0.000***^†^ | 15.52 | 0.001**^†^ | 14.60 | 0.001**^†^ |

Notes: U-C = pattern similarity between Uyghur and Chinese words.

U-E = pattern similarity between Uyghur and English words.

**^a^**The comparisons of pattern similarity between U-C and U-E.

^b^The comparisons of pattern similarity between U-C and U-E while controlling for covariates (i.e., the differences in age of acquisition between Chinese and English).

^c^The comparisons of pattern similarity between U-C and U-E while controlling for covariates (i.e., the differences in age of acquisition between Uyghur and Chinese, Uyghur and English)

**p* < 0.05, ***p* < 0.01 and ****p* < 0.001, ^†^*p* < 0.005 (significance level after Bonferroni correction).

**Table S6 Stimuli of the three languages used in this study.**

| **Uyghur words** | | | **Chinese words** | | | **English words** | | |
| --- | --- | --- | --- | --- | --- | --- | --- | --- |
| Orthography | Phonology | Semantics | Orthography | Phonology | Semantics | Orthography | Phonology | Semantics |
| قەغەز | [kæᴚæz] | paper | 井 | [jǐng] | well | bag | [bæɡ] | bag |
| ئۈزۈم | [yzym] | grapes | 尺 | [chǐ] | ruler | friend | [frend] | friend |
| چوكا | [tʃoka] | chopsticks | 枣 | [zǎo] | dates | gas | [ɡæs] | gas |
| ئانا | [ana] | mother | 库 | [kù] | warehouse | adult | [ˈædʌlt] | adult |
| خەت | [xæt] | letter | 瓜 | [guā] | melon | age | [eɪdʒ] | age |
| توك | [tok] | electricity | 贝 | [bèi] | shell | girl | [ɡɜːl] | girl |
| ئىشىك | [iʃik] | door | 伯 | [bó] | uncle | advice | [ədˈvaɪs] | advice |
| سائەت | [saæt] | watch | 仓 | [cāng] | granary | cancer | [ˈkænsə(r)] | cancer |
| ياڭاق | [jaŋak] | walnut | 鸡 | [jī] | chicken | hero | [ˈhɪərəʊ] | hero |
| مۇز | [muz] | ice | 炉 | [lú] | furnace | answer | [ˈɑːnsə(r)] | answer |
| بوۋا | [buva] | grandpa | 壳 | [ké] | shuck | art | [ɑːt] | art |
| گۆش | [gθʃ] | meat | 卡 | [kǎ] | card | dancer | [ˈdɑːnsə(r)] | dancer |
| كۆز | [kθz] | eye | 汁 | [zhī] | sap | cake | [keɪk] | cake |
| قۇياش | [qujaʃ] | sun | 狐 | [hú] | fox | food | [fuːd] | food |
| ئەينەك | [æjnæk] | mirror | 斧 | [fǔ] | axe | egg | [eɡ] | egg |
| تىلفۇن | [tilfon] | phone | 册 | [cè] | volume | animal | [ˈænɪml] | animal |
| چۆچۈرە | [tʃθtʃyræ] | wonton | 豆 | [dòu] | bean | doctor | [ˈdɒktə(r)] | doctor |
| ئويۇن | [oyjun] | game | 肠 | [cháng] | intestines | death | [deθ] | death |
| يەر | [jær] | land | 凤 | [fèng] | phoenix | farm | [fɑːm] | farm |
| قول | [qol] | hand | 肌 | [jī] | muscle | law | [lɔː] | law |
| ئشچى | [iʃtʃi] | workers | 园 | [yuán] | garden | brain | [breɪn] | brain |
| پۇل | [pul] | money | 网 | [wǎng] | net | beach | [biːtʃ] | beach |
| تاۋۇز | [tawuz] | watermelon | 帆 | [fān] | sail | glass | [ɡlɑːs] | glass |
| يۈز | [jyz] | face | 汤 | [tāng] | soup | lady | [ˈleɪdi] | lady |
| دەريا | [dærja] | river | 杯 | [bēi] | cup | foot | [fʊt] | foot |
| شاپتۇل | [ʃaptul] | peach | 虎 | [hǔ] | tiger | juice | [dʒuːs] | juice |
| تۈگمە | [tygmæ] | button | 泪 | [lèi] | tear | bank | [bæŋk] | bank |
| يامغۇر | [jamᴚur] | rain | 肚 | [dù] | stomach | camera | [ˈkæmərə] | camera |
| شام | [ʃam] | candle | 瓦 | [wǎ] | tile | aim | [eɪm] | aim |
| لۆڭگە | [lθŋgæ] | towel | 龟 | [guī] | turtle | heart | [hɑːt] | heart |
| گۈل | [gyl] | flower | 谷 | [gǔ] | grain | act | [ækt] | act |
| قاش | [qaʃ] | eyebrow | 词 | [cí] | word | fish | [fɪʃ] | fish |
| پىل | [pil] | elephant | 苗 | [miáo] | seedling | idea | [aɪˈdɪə] | idea |
| ئۈزۈك | [yzuk] | ring | 茄 | [qié] | eggplant | fan | [fæn] | fan |
| دوسكا | [doska] | blackboard | 波 | [bō] | wave | candy | [ˈkændi] | candy |
| نەشپۈت | [næʃpyt] | pear | 耳 | [ěr] | ear | gun | [ɡʌn] | gun |
| ناخشا | [naxʃa] | song | 庙 | [miào] | temple | habit | [ˈhæbɪt] | habit |
| چاچ | [tʃatʃ] | hair | 妻 | [qī] | wife | book | [bʊk] | book |
| رەسىم | [ræsim] | photo | 兄 | [xiōng] | brother | birth | [bɜːθ] | birth |
| تام | [tam] | wall | 君 | [jūn] | gentleman | farmer | [ˈfɑːmə(r)] | farmer |
| يول | [jol] | road | 云 | [yún] | cloud | author | [ˈɔːθə(r)] | author |
| چىش | [tʃiʃ] | teeth | 肺 | [fèi] | lung | dream | [driːm] | dream |
| ماشىنا | [maʃina] | car | 玉 | [yù] | jade | bee | [biː] | bee |
| بارماق | [barmak] | finger | 岛 | [dǎo] | island | boy | [bɔɪ] | boy |
| قار | [qar] | snow | 乳 | [rǔ] | breast | hat | [hæt] | hat |
| ئاچقۇچ | [atʃqutʃ] | key | 肩 | [jiān] | shoulder | health | [helθ] | health |
| گىلەم | [gilæm] | carpet | 帐 | [zhàng] | tent | gift | [ɡɪft] | gift |
| ساقچى | [saqtʃi] | police | 驴 | [lǘ] | donkey | bed | [bed] | bed |
| دەپتەر | [dæptær] | notebook | 厕 | [cè] | toilet | box | [bɒks] | box |
| بالا | [bala] | child | 诗 | [shī] | poetry | bottle | [ˈbɒtl] | bottle |
| ئىسىم | [isim] | name | 兔 | [tù] | rabbit | dress | [dres] | dress |
| دورا | [dora] | medicine | 羊 | [yáng] | sheep | image | [ˈɪmɪdʒ] | image |
| مەيدان | [mæjdan] | playground | 龙 | [lóng] | dragon | gold | [ɡəʊld] | gold |
| بويۇن | [bojun] | neck | 宅 | [zhái] | residence | human | [ˈhjuːmən] | human |
| رەخ | [ræx] | cloth | 岩 | [yán] | rock | fruit | [fruːt] | fruit |
| تاغ | [taᴚ] | mountain | 肾 | [shèn] | renal | beef | [biːf] | beef |
| پولو | [polo] | meal | 礼 | [lǐ] | ceremony | line | [laɪn] | line |
| سافا | [safa] | sofa | 池 | [chí] | pool | joke | [dʒəʊk] | joke |
| سىنىپ | [sinip] | classroom | 佛 | [fó] | buddha | fly | [flaɪ] | fly |
| پۇتبول | [putbol] | football | 戏 | [xì] | drama | jacket | [ˈdʒækɪt] | jacket |
| سۈت | [syt] | milk | 芽 | [yá] | bud | boss | [bɒs] | boss |
| ئاياغ | [ajaᴚ] | shoe | 钉 | [dīng] | nail | body | [ˈbɒdi] | body |
| تىرناق | [tirnaq] | fingernail | 帘 | [lián] | curtain | earth | [ɜːθ] | earth |
| يۇلتۇز | [jultuz] | star | 妹 | [mèi] | sister | lemon | [ˈlemən] | lemon |
| كىنو | [kino] | movie | 汗 | [hàn] | sweat | lake | [leɪk] | lake |
| پىچىنە | [pitʃinæ] | cookies | 奴 | [nú] | slave | desk | [desk] | desk |
| نان | [nan] | pancakes | 丝 | [sī] | silk | air | [eə(r)] | air |
| تورت | [tort] | cake | 妖 | [yāo] | monster | dinner | [ˈdɪnə(r)] | dinner |
| ئاۋاز | [awaz] | voice | 伞 | [sǎn] | umbrella | job | [dʒəʊb] | job |
| مەكتەپ | [mæktæp] | school | 竹 | [zhú] | bamboo | driver | [ˈdraɪvə(r)] | driver |
| مۇزىكا | [muzika] | music | 肝 | [gān] | liver | floor | [flɔː(r)] | floor |
| ھەرە | [hæræ] | bee | 杏 | [xìng] | apricot | dog | [dɒɡ] | dog |
| ھىكايە | [hikajæ] | story | 卵 | [luǎn] | egg | hotel | [həʊˈtel] | hotel |
| ئەتىىر | [ætir] | perfume | 舌 | [shé] | tongue | coffee | [ˈkɒfi] | coffee |
| زەنجىر | [zændʒir] | necklace | 麦 | [mài] | wheat | hour | [ˈaʊə(r)] | hour |
| مۈشۈك | [myʃyk] | cat | 刺 | [cì] | thorn | leg | [leɡ] | leg |
| قۇلۇپ | [qulup] | lock | 厅 | [tīng] | hall | horse | [hɔːs] | horse |
| دادا | [dada] | father | 茎 | [jīng] | stem | lesson | [ˈlesn] | lesson |
| شارپا | [ʃarpa] | scarf | 财 | [cái] | money | knife | [naɪf] | knife |
| ئىشتان | [iʃtan] | trousers | 枕 | [zhěn] | pillow | ill | [ɪl] | ill |

**Table S7 The comparisons of cross-language pattern similarity in the 22 brain regions for comprehensive word processing.**

| **ROIs** | | **U-C** | **U-E** | **F^a^** | **P^a^** |
| --- | --- | --- | --- | --- | --- |
|  |  | **M** | **M** |  |  |
| **Left** |  |  |  |  |  |
|  | Pars triangularis | 1.06 | 1.26 | 25.69 | 0.000***^†^ |
|  | Frontal orbital cortex | 1.15 | 1.23 | 8.70 | 0.007** |
|  | Pars opercularis | 1.14 | 1.30 | 17.98 | 0.000***^†^ |
|  | Precentral gyrus | 1.40 | 1.55 | 17.14 | 0.000***^†^ |
|  | Superior temporal gyrus | 1.36 | 1.48 | 40.00 | 0.000***^†^ |
|  | Middle temporal gyrus | 1.13 | 1.26 | 28.76 | 0.000***^†^ |
|  | Inferior temporal gyrus | 1.09 | 1.17 | 19.39 | 0.000***^†^ |
|  | Fusiform gyrus | 1.21 | 1.35 | 21.79 | 0.000***^†^ |
|  | Supramarginal gyrus | 1.11 | 1.28 | 16.66 | 0.000***^†^ |
|  | Superior parietal lobule | 1.13 | 1.29 | 16.81 | 0.000***^†^ |
|  | Angular gyrus | 1.09 | 1.22 | 12.05 | 0.002** |
| **Right** |  |  |  |  |  |
|  | Pars triangularis | 1.13 | 1.22 | 5.42 | 0.030* |
|  | Frontal orbital cortex | 1.12 | 1.23 | 11.85 | 0.002** |
|  | Pars opercularis | 1.15 | 1.30 | 29.48 | 0.000***^†^ |
|  | Precentral gyrus | 1.48 | 1.58 | 8.78 | 0.007** |
|  | Superior temporal gyrus | 1.43 | 1.53 | 24.74 | 0.000***^†^ |
|  | Middle temporal gyrus | 1.29 | 1.40 | 29.81 | 0.000***^†^ |
|  | Inferior temporal gyrus | 1.12 | 1.18 | 4.77 | 0.040* |
|  | Fusiform gyrus | 1.30 | 1.31 | 0.04 | 0.852 |
|  | Supramarginal gyrus | 1.18 | 1.30 | 5.30 | 0.031* |
|  | Superior parietal lobule | 1.18 | 1.29 | 9.80 | 0.005** |
|  | Angular gyrus | 1.07 | 1.19 | 18.40 | 0.000***^†^ |

Note：U-C = pattern similarity between Uyghur and Chinese words.

U-E = pattern similarity between Uyghur and English words.

**^a^**The comparisons of pattern similarity between U-C and U-E.

**p* < 0.05, ***p* < 0.01 and ****p* < 0.001, ^†^*p* < 0.002.

**Table S8 Spearman correlations between cross-language neural dissimilarity matrices and the three prediction matrices in the 13 brain regions showing significant effects in Table S7.**

| Brain Regions | Visual | | Phonological | | Semantic | | Phonological  **(adjusted)** | |
| --- | --- | --- | --- | --- | --- | --- | --- | --- |
|  | r | *p* | r | *p* | r | *p* | r | *p* |
| *Uyghur-English* |  |  |  |  |  |  |  |  |
| Left pars triangularis | -0.006 | 0.239 | 0.013 | 0.000*** | 0.002 | 0.487 | 0.013 | 0.000*** |
| Left pars opercularis | 0.005 | 0.359 | 0.010 | 0.004** | 0.000 | 0.971 | 0.009 | 0.004** |
| Right pars opercularis | 0.008 | 0.118 | 0.014 | 0.000*** | 0.001 | 0.683 | 0.013 | 0.000*** |
| Left precentral gyrus | 0.000 | 0.958 | 0.014 | 0.000*** | 0.002 | 0.542 | 0.014 | 0.001** |
| Left superior temporal gyrus | 0.005 | 0.393 | 0.012 | 0.000*** | 0.005 | 0.230 | 0.012 | 0.000*** |
| Right superior temporal gyrus | 0.009 | 0.078 | 0.016 | 0.000*** | 0.004 | 0.351 | 0.016 | 0.000*** |
| Left middle temporal gyrus | 0.001 | 0.830 | 0.013 | 0.001** | 0.001 | 0.806 | 0.013 | 0.001** |
| Right middle temporal gyrus | 0.003 | 0.599 | 0.014 | 0.000*** | 0.003 | 0.488 | 0.014 | 0.000*** |
| Left inferior temporal gyrus | -0.005 | 0.473 | 0.010 | 0.003** | 0.005 | 0.154 | 0.010 | 0.003** |
| Left fusiform gyrus | 0.008 | 0.126 | 0.013 | 0.001** | 0.004 | 0.293 | 0.013 | 0.001** |
| Left supramarginal gyrus | 0.002 | 0.764 | 0.011 | 0.002** | 0.005 | 0.167 | 0.011 | 0.002** |
| Left superior parietal lobule | 0.003 | 0.677 | 0.011 | 0.001** | 0.009 | 0.013* | 0.011 | 0.001** |
| Right angular gyrus | 0.008 | 0.089 | 0.012 | 0.001** | 0.006 | 0.161 | 0.012 | 0.001** |
|  |  |  |  |  |  |  |  |  |
| *Uyghur-Chinese* |  |  |  |  |  |  |  |  |
| Left pars triangularis | 0.012 | 0.078 | 0.003 | 0.374 | 0.006 | 0.090 | 0.003 | 0.374 |
| Left pars opercularis | 0.001 | 0.944 | 0.001 | 0.707 | 0.001 | 0.588 | 0.001 | 0.706 |
| Right pars opercularis | 0.007 | 0.308 | 0.006 | 0.085 | 0.001 | 0.845 | 0.006 | 0.086 |
| Left precentral gyrus | 0.010 | 0.128 | 0.004 | 0.115 | -0.001 | 0.717 | 0.004 | 0.119 |
| Left superior temporal gyrus | 0.014 | 0.076 | 0.003 | 0.358 | 0.000 | 0.867 | 0.003 | 0.371 |
| Right superior temporal gyrus | 0.009 | 0.225 | 0.005 | 0.143 | 0.002 | 0.506 | 0.005 | 0.146 |
| Left middle temporal gyrus | 0.002 | 0.535 | 0.002 | 0.517 | -0.002 | 0.312 | 0.002 | 0.535 |
| Right middle temporal gyrus | 0.015 | 0.086 | -0.001 | 0.814 | -0.002 | 0.546 | -0.001 | 0.798 |
| Left inferior temporal gyrus | 0.009 | 0.236 | 0.004 | 0.291 | -0.002 | 0.397 | 0.004 | 0.299 |
| Left fusiform gyrus | 0.010 | 0.116 | 0.004 | 0.197 | -0.003 | 0.194 | 0.004 | 0.204 |
| Left supramarginal gyrus | 0.007 | 0.417 | 0.002 | 0.597 | -0.003 | 0.331 | 0.002 | 0.610 |
| Left superior parietal lobule | 0.009 | 0.349 | 0.004 | 0.291 | -0.002 | 0.521 | 0.004 | 0.299 |
| Right angular gyrus | 0.006 | 0.362 | -0.002 | 0.589 | -0.002 | 0.461 | -0.002 | 0.580 |

Notes: Phonological (adjusted) represents partial correlation between neural dissimilarity matrix and phonological prediction matrix after controlling for visual and semantic prediction matrices.**p* < 0.05, ***p* < 0.01, and ****p* < 0.001.


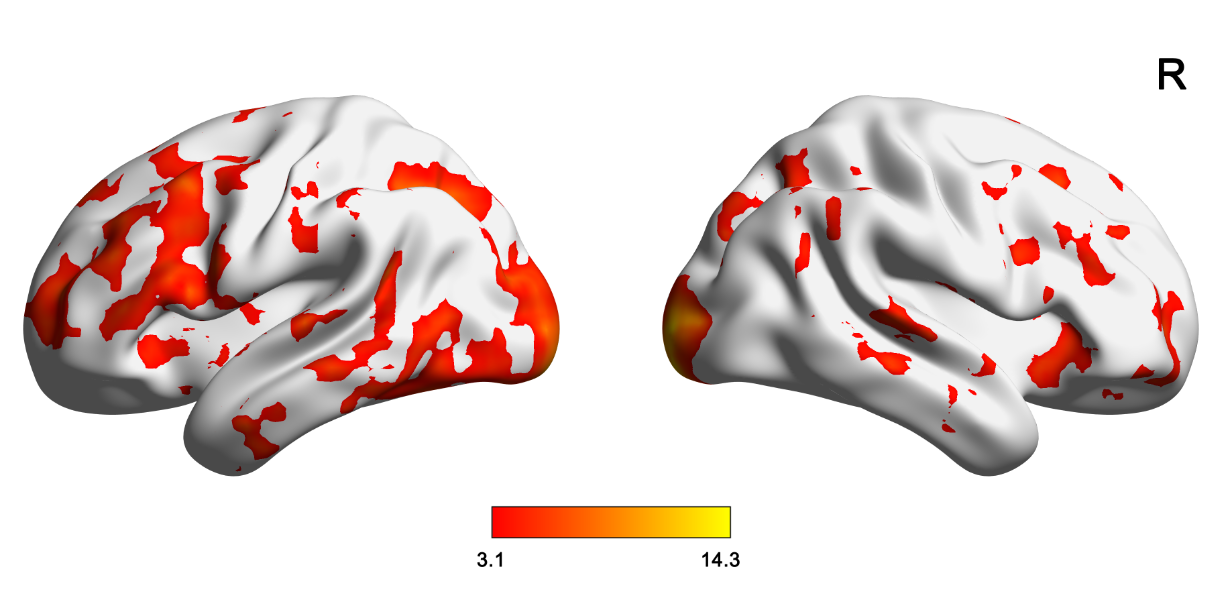


**Figure S1.** Brain maps for representational similarity analysis after controlling for the differences in reaction time between Chinese and English words. It presents brain regions showing greater pattern similarity between Uyghur and English than that between Uyghur and Chinese. All activations were thresholded at Z > 3.1 (whole-brain corrected). R = right.





**Figure S2.** The three prediction matrices for each cross-language pair (i.e., Uyghur-English and Uyghur-Chinese). For the visual prediction matrices (A), a binary silhouette of each word was used to compute the pixel-wise non-overlap regions of the two images in each cross-language pair. For the phonological prediction matrices (B), we used the second coding scheme from the MatchCalculator tool, which was developed by Colin Davis [(www.pc.rhul.ac.uk/staff/c.davis/Utilities/MatchCalc/)](../Response%20to%20Reviews-2020.2.10.doc). It was calculated as 1 minus the proportion of same-position phonemes shared across the two words in each cross-language pair. The semantic prediction matrices (C) were estimated by dividing the words in the three languages into twelve categories according to their semantic similarity. Item pairs from the same semantic category were denoted as 0, and pairs from different categories were denoted as 1.


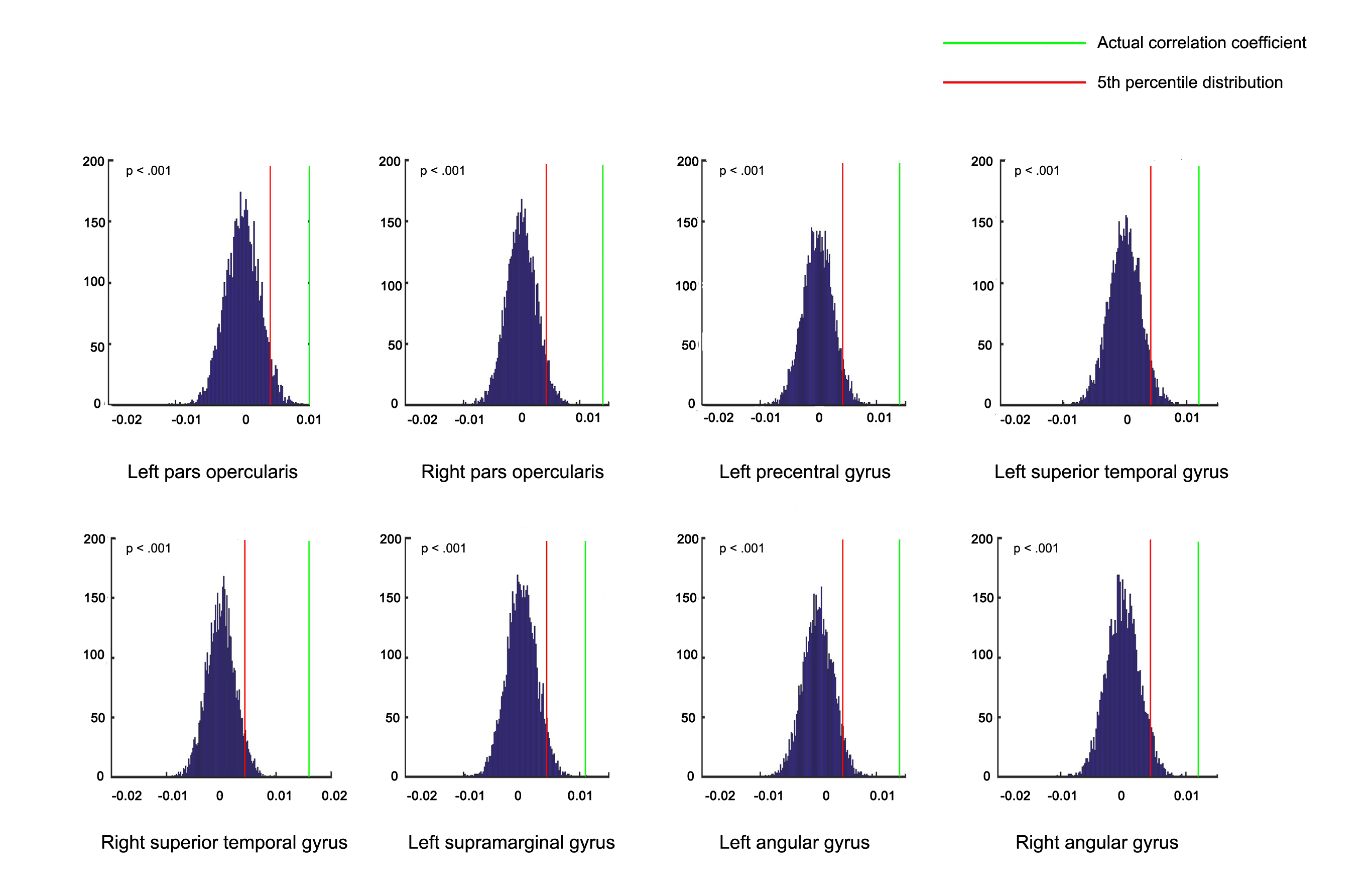


**Figure S3.** The histogram plots of permutation test in the 8 ROIs which showed significant correlations between cross-language neural dissimilarity matrix and phonological prediction matrix. The green line indicates the actual correlation between neural dissimilarity matrix and phonological prediction matrix for all Uyghur-English item pairs, and the red line indicates the 5th percentile (0.05) of the distribution. X-axis represents the correlation coefficients.


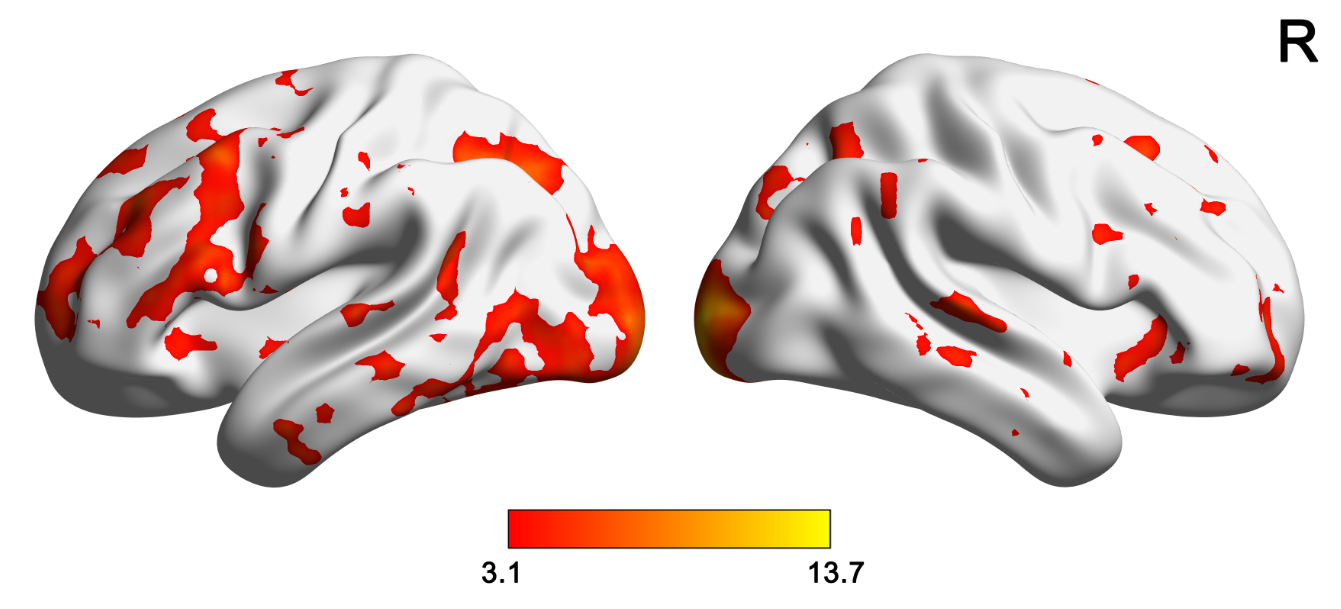


**Figure S4.** Brain maps for representational similarity analysis after controlling for the differences in age of acquisition between Chinese and English. It presents brain regions showing greater pattern similarity between Uyghur and English than that between Uyghur and Chinese. All activations were thresholded at Z > 3.1 (whole-brain corrected). R = right.
